# Supplementary material for: Identification of Freezing-Responsive microRNAs and Their Targets in Chinese Jujube by Small RNA and Degradome Sequencing
Source: Int J Mol Sci. 2026 Sep 4;27(17):7912. doi: 10.3390/ijms27177912 (PMC13566890; doi:10.3390/ijms27177912)
Supplement: Supplementary file 1 [file ijms-27-07912-s001.zip › Supplementary Material S2/novel_miR_13.pdf]

Provisional ID : novel_miR_13
Score total : 106160.7
Score for star read(s) : 3.9
Score for read counts : 106154.4
Score for mfe : 0.8
Score for randfold : 1.6
Score for cons. seed :
Total read count : 208229
Mature read count : 191761
Loop read count : 9
Star read count : 16459

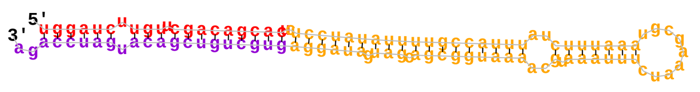

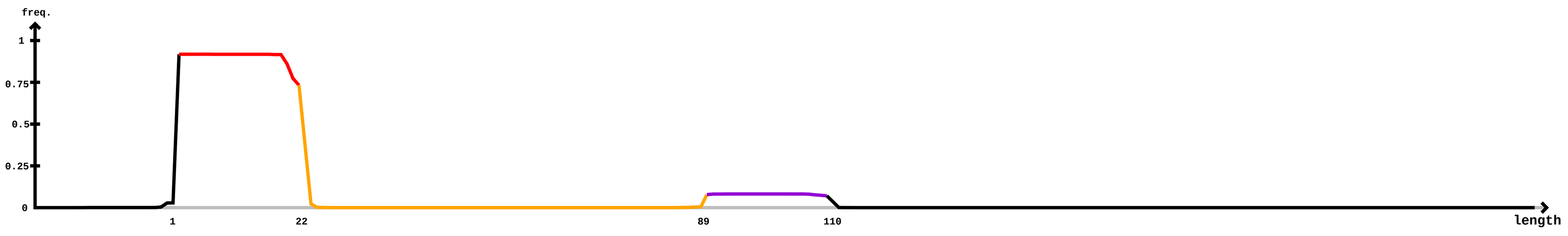


```
               Mature                                                                                    Star
5'- uccaucuuguucguuuacccucuggaucuuguucgacagcacucuccuauauuuugccauuuaucuuuaaaugcgaaaucuuuaaaugcaaaauggcgacgaugauaggagugcugucgacaugauccagaaggugcagucgaauaagauacagagggaccacccacaguaccuacucucaacucucaucgcucgcaaucuuauucuucuccucacuuauuauuuuucagaucauuuucuuuuaaucuau  -3'  obs
    uccaucuuguucguuuacccucuggaucuuguucgacagcacucuccuauauuuugccauuuaucuuuaaaugcgaaaucuuuaaaugcaaaauggcgacgaugauaggagugcugucgacaugauccagaaggugcagucgaauaagauacagagggaccacccacaguaccuacucucaacucucaucgcucgcaaucuuauucuucuccucacuuauuauuuuucagaucauuuucuuuuaaucuau       exp
    ...((((((((((((((.((((((((.(((.(((((((((..((((((((((((((((((..(((((((........))))))).)..)))))))))).))).))))))))))))))))))).)))))))).)))).)).))))))))))..((((((((........)).))..))))..............................................(((......)))..........  reads  mm  sample
    .ccaucuuguucguuuacc.......................................................................................................................................................................................................................................  1  0  LT-1
    .ccaucuuguucguuuacccucugg.................................................................................................................................................................................................................................  5  0  LCK-3
    .ccaucuuguucguuuacccucuggauc..............................................................................................................................................................................................................................  1  0  LT-3
    ...aucuuguucguuuacccucugga................................................................................................................................................................................................................................  2  0  JCK-1
    .....cuuguucguuuacccucuggauc..............................................................................................................................................................................................................................  4  0  LT-2
    ......uuguucguuuaccUucuggauc..............................................................................................................................................................................................................................  2  1  LT-2
    ......uuguucguuuaccUucuggauc..............................................................................................................................................................................................................................  3  1  JCK-3
    ......uuguucguuuaccUucuggauc..............................................................................................................................................................................................................................  2  1  JCK-1
    ......uuguucguuuaccUucuggauc..............................................................................................................................................................................................................................  6  1  JCK-2
    .......uguucguuuacccucuggauc..............................................................................................................................................................................................................................  1  0  JCK-1
    .......uguucguuuaccUucuggauc..............................................................................................................................................................................................................................  3  1  LT-2
    .......uguucguuuacccucuggauc..............................................................................................................................................................................................................................  1  0  JCK-2
    .......uguucguuuaccUucuggauc..............................................................................................................................................................................................................................  12  1  LT-3
    .......uguucguuuacccucuggauc..............................................................................................................................................................................................................................  2  0  JCK-3
    .......uguucguuuaccUucuggauc..............................................................................................................................................................................................................................  31  1  JCK-2
    .......uguucguuuaccUucuggauc..............................................................................................................................................................................................................................  12  1  LT-1
    .......uguucguuuaccUucuggauc..............................................................................................................................................................................................................................  10  1  JCK-1
    .......uguucguuuaccUucuggauc..............................................................................................................................................................................................................................  30  1  JCK-3
    ..........ucguuuacccucuggaucuug...........................................................................................................................................................................................................................  1  0  LT-2
    .............uuuacccucuggaucuuguA.........................................................................................................................................................................................................................  1  1  JCK-1
    .............uuuacccucuggaucuuguuc........................................................................................................................................................................................................................  1  0  JCK-2
    .............uuuacccucuggaucuuguuc........................................................................................................................................................................................................................  1  0  JCK-3
    ..................ccucuggaucuuguucgaca....................................................................................................................................................................................................................  1  0  JCK-1
    ..................ccucuggaucuuguucgacag...................................................................................................................................................................................................................  5  0  LT-3
    ..................ccucuggaucuuguucgacagc..................................................................................................................................................................................................................  8  0  JCK-2
    ..................ccucuggaucuuguucgacagc..................................................................................................................................................................................................................  4  0  JCK-3
    ..................ccucuggaucuuguucgacagc..................................................................................................................................................................................................................  2  0  LT-1
    ..................ccucuggaucuuguucgacagc..................................................................................................................................................................................................................  6  0  JCK-1
    ..................ccucuggaucuuguucgacagc..................................................................................................................................................................................................................  2  0  LT-3
    ..................ccucuggaucuuguucgacagc..................................................................................................................................................................................................................  2  0  LT-2
    ..................ccucuggCucuuguucgacagcacu...............................................................................................................................................................................................................  1  1  JCK-2
    ..................ccucuggaucuuguucgacagcacu...............................................................................................................................................................................................................  1  0  JCK-2
    ..................ccucuggaucuuguucgacagcacu...............................................................................................................................................................................................................  1  0  JCK-3
```

```
                      Mature                                                                      Star
uccaucuuguucguuuacccucuggaucuuguucgacagcacucuccuauauuuugccauuuaucuuuaaaugcgaaaucuuuaaaugcaaaauggcgacgaugauaggagugcugucgacaugauccagaaggugcagucgaauaagauacagagggaccacccacaguaccuacucucaacucucaucgcucgcaaucuuauucuucuccucacuuauuauuuuucagaucauuuucuuuuaaucuau
..................ccucuggaucuuguucgacagcacu      1    0   JCK-1
...................cucuggaucuuguucgac            1    0   LT-3
...................cucuggaucuuguucgaca           6    0   LT-3
...................cucuggaucuuguucgaca           2    0   LT-1
...................cucuggaucuuguucgaca           8    0   LCK-3
...................cucuggaucuuguucgaca           6    0   LT-2
...................cucuggaucuuguucgacagc         43   0   JCK-2
...................cucuggaucuuguucgacagc         5    0   LCK-3
...................cCcuggaucuuguucgacagc         2    1   LT-1
...................cucuggaucuuguucgacagc         8    0   LT-2
...................cucuggaucuuguucgacagc         20   0   LT-1
...................cucuggaucuuguucgacagc         36   0   JCK-1
...................cucuggCucuuguucgacagc         1    1   JCK-2
...................cucuggaucuuguucgacagc         34   0   JCK-3
...................cucuggaucuuguucgacagc         22   0   LT-3
...................cNcuggaucuuguucgacagca        1    1   JCK-2
...................cuUuggaucuuguucgacagca        1    1   JCK-3
...................cucuggaucuuguucgaAagca        1    1   JCK-1
...................cucuggaucuuguucgacagcG        1    1   JCK-1
...................cucuggaucuuguucgacagca        24   0   LCK-3
...................cucuggaucuuguucgacagUa        2    1   JCK-2
...................cucuggaucuuguucgacaNca        1    1   JCK-1
...................cucuggaucuuNuucgacagca        1    1   JCK-1
...................cucuggaucuuguucgacagca        137  0   JCK-2
...................cucuggaucuuguucgacagca        103  0   JCK-3
...................cucuggaucuuguucgacagcU        1    1   JCK-1
...................cucuggaucuuguucgacagcU        3    1   JCK-2
...................cNcuggaucuuguucgacagca        1    1   JCK-3
...................cucuggaucuuguucgacagca        81   0   JCK-1
...................Aucuggaucuuguucgacagca        2    1   JCK-3
...................cucuggaucuuguucgacagca        12   0   LT-3
...................cucuggaucuuguucgacCgca        1    1   JCK-1
...................cucuggaucuuguucgCcagca        1    1   LT-1
...................cucuggaucuuguucgacagca        19   0   LT-1
...................cucuggaucuuguucgacagca        6    0   LT-2
...................cucuggaucuuguucgacagcacu      1    0   JCK-3
...................cucuggaucuuguucgacagcacucu    1    0   LT-3
....................ucuggaucuuguucgacU           2    1   LT-1
....................ucuggaucuuguucgaca           14   0   JT-2
....................ucuggaucuuguucgacU           2    1   LCK-3
....................ucuggaucuuguucgCca           1    1   LT-3
....................ucuggaucuuguucgaca           23   0   JCK-2
....................ucuggaucuuguucgaca           17   0   JT-3
....................ucuggaucuuguucgaca           53   0   LT-3
....................ucuggaucuuguucgacU           1    1   JCK-1
....................ucuggaucuuguucgaca           12   0   JT-1
....................ucuggaucuuguucgaca           17   0   LT-1
....................ucuggaucuuguucgaca           37   0   LT-2
....................ucuggaucuuguucgacU           1    1   LT-3
....................ucuggaucuuguucgacU           1    1   JCK-2
....................ucuggaucuuguucgaca           22   0   JCK-3
....................ucuggaucuuguucgaca           76   0   LCK-3
....................ucuggaucuuguucgaca           25   0   JCK-1
....................ucuggaucuuguucgacaU          2    1   JCK-2
....................ucuggaucuuguucgacag          1    0   JCK-1
....................ucuggaucuuguucgacaA          1    1   LT-1
....................ucuggaucuuguucgacag          1    0   LT-3
....................ucuggaucuuguucgacaU          2    1   JCK-3
....................ucuggNucuuguucgacagc         1    1   LCK-1
....................ucuggCucuuguucgacagc         1    1   LT-1
....................ucuggaucuuguucgacagU         1    1   LT-1
....................ucuggaucuuguucgacagc         33   0   JCK-1
....................ucuggaucuuguucgacagc         5    0   JT-2
....................ucuggaucuuguucgacagc         69   0   LT-2
....................ucuggaucuuguucgacagc         20   0   LCK-1
....................ucuggaucuuguucgacagc         56   0   JCK-2
....................ucuggaucuuguucgacagc         4    0   JT-3
....................ucuggaucuuguucgacagc         52   0   LT-1
....................ucuggaucuuguucgacagc         28   0   LCK-2
....................ucuggaucuuguucgacagc         29   0   JCK-3
```

Mature   Star

```
uccaucuuguucguuuacccucuggaucuuguucgacagcacucuccuauauuuugccauuuaucuuuaaaugcgaaaucuuuaaaugcaaaauggcgacgaugauaggagugcugucgacaugauccagaaggugcagucgaauaagauacagagggaccacccacaguaccuacucucaacucucaucgcucgcaaucuuauucuucuccucacuuauuauuuuucagaucauuuucuuuuaaucuau
....................ucuggaucuuguucgacagc..................................................................................................................................................................................   80     0    LT-3
....................ucuggaucuuguucgacagc..................................................................................................................................................................................   62     0    LCK-3
....................ucuggaucuuguucgaAagca.................................................................................................................................................................................   1      1    JCK-2
....................ucuggaucuuguucgCcagca.................................................................................................................................................................................   1      1    JT-1
....................ucuggaucuuguucgacagca.................................................................................................................................................................................   83     0    LT-2
....................ucuggaucuuguucgacagca.................................................................................................................................................................................   128    0    LT-3
....................ucuggaucuuguucgacagcU.................................................................................................................................................................................   3      1    JCK-3
....................ucuggaucuuguucgacagca.................................................................................................................................................................................   1162   0    JCK-3
....................ucuggaucuuguucgacagcU.................................................................................................................................................................................   8      1    JCK-2
....................ucuggaucuuguucgacagca.................................................................................................................................................................................   19     0    JT-3
....................ucuggaucuuguucgacagca.................................................................................................................................................................................   31     0    LCK-2
....................ucCggaucuuguucgacagca.................................................................................................................................................................................   1      1    JCK-3
....................ucuggaucuuguucgacagca.................................................................................................................................................................................   9      0    JT-2
....................ucuggaucCuguucgacagca.................................................................................................................................................................................   1      1    LT-2
....................ucuggaucuuguucgaAagca.................................................................................................................................................................................   1      1    JCK-1
....................ucuggaucCuguucgacagca.................................................................................................................................................................................   1      1    JCK-2
....................ucuggaucuugNucgacagca.................................................................................................................................................................................   2      1    JCK-1
....................ucuggaucuuguucgacagca.................................................................................................................................................................................   56     0    JT-1
....................ucuggauAuuguucgacagca.................................................................................................................................................................................   1      1    JCK-3
....................ucuggaucuuguucgacagcU.................................................................................................................................................................................   2      1    LT-3
....................ucuggaucuuguucgacaUca.................................................................................................................................................................................   1      1    JCK-1
....................uUuggaucuuguucgacagca.................................................................................................................................................................................   5      1    JCK-3
....................ucuggaucuuguucgacagca.................................................................................................................................................................................   109    0    LT-1
....................Ccuggaucuuguucgacagca.................................................................................................................................................................................   1      1    JCK-2
....................ucuggaucuuguucgaAagca.................................................................................................................................................................................   1      1    JCK-3
....................ucuggaucuuguucgacagca.................................................................................................................................................................................   882    0    JCK-1
....................ucuggaucuuguucgCcagca.................................................................................................................................................................................   1      1    JCK-1
....................ucuggaucuuguucgCcagca.................................................................................................................................................................................   1      1    LT-1
....................Ccuggaucuuguucgacagca.................................................................................................................................................................................   1      1    LT-3
....................ucuggaucuuguucgacagca.................................................................................................................................................................................   27     0    LCK-1
....................ucuggaucuuguucgaUagca.................................................................................................................................................................................   1      1    JCK-3
....................ucuggaucuuguucgacagUa.................................................................................................................................................................................   1      1    JCK-3
....................ucuggaucuuguucgacagcU.................................................................................................................................................................................   3      1    LCK-3
....................ucuggCucuuguucgacagca.................................................................................................................................................................................   1      1    JCK-3
....................ucuggaucuuguucgacagcU.................................................................................................................................................................................   2      1    JCK-1
....................ucuggaucuuguucAacagca.................................................................................................................................................................................   1      1    JCK-1
....................uNuggaucuuguucgacagca.................................................................................................................................................................................   2      1    JCK-2
....................uUuggaucuuguucgacagca.................................................................................................................................................................................   5      1    JCK-1
....................ucuggaucuuguucgacagcN.................................................................................................................................................................................   1      1    JCK-1
....................ucuggaucuuguucgacaUca.................................................................................................................................................................................   3      1    JCK-2
....................ucugAaucuuguucgacagca.................................................................................................................................................................................   1      1    JCK-1
....................ucuggaucuuguucgacagcC.................................................................................................................................................................................   1      1    JCK-1
....................ucuggaucuuguucgacagca.................................................................................................................................................................................   1463   0    JCK-2
....................ucuggCucuuguucgacagca.................................................................................................................................................................................   4      1    JCK-2
....................ucuggaucuuguucgacagca.................................................................................................................................................................................   76     0    LCK-3
....................ucuggaucuuguucgacagcU.................................................................................................................................................................................   3      1    LT-2
....................uUuggaucuuguucgacagca.................................................................................................................................................................................   5      1    JCK-2
....................ucuggaucuuguucgacaAca.................................................................................................................................................................................   1      1    JCK-2
....................ucNggaucuuguucgacagca.................................................................................................................................................................................   1      1    LCK-1
....................ucuggaucuuguucgacagcac................................................................................................................................................................................   7      0    LT-2
....................ucuggaucuuguucgacagcac................................................................................................................................................................................   16     0    LCK-3
....................ucuggaucuuguucgacagcac................................................................................................................................................................................   10     0    LT-1
....................ucuggaucuuguucgacagcac................................................................................................................................................................................   16     0    JCK-1
....................ucuggaucuuguucgacagcac................................................................................................................................................................................   37     0    JCK-2
....................ucuggaucuuguucgacagcac................................................................................................................................................................................   30     0    JCK-3
....................ucuggaucuuguucgacagcaA................................................................................................................................................................................   1      1    JCK-3
....................ucuggaucuuguucgacagcac................................................................................................................................................................................   11     0    LT-3
....................ucuggaucuuguucgacagcacA...............................................................................................................................................................................   1      1    JCK-2
....................ucuggaucuuguucgacagcacA...............................................................................................................................................................................   2      1    JCK-1
....................ucuggaucuuguucgacagcacu...............................................................................................................................................................................   3      0    LT-3
....................ucuggaucuuguucgacagcacu...............................................................................................................................................................................   8      0    LCK-3
....................ucuggaucuuguucgacagcaAu...............................................................................................................................................................................   3      1    LT-3
....................ucuggaucuuguucgacagcacu...............................................................................................................................................................................   4      0    JCK-1
....................ucuggaucuuguucgacagcacA...............................................................................................................................................................................   1      1    JCK-3
....................ucuggaucuuguucgacagcacu...............................................................................................................................................................................   2      0    LT-2
....................ucuggaucuuguucgacagcacu...............................................................................................................................................................................   9      0    JCK-2
....................ucuggaucuuguucgacagcacC...............................................................................................................................................................................   1      1    LT-3
....................ucuggaucuuguucgacagcaUu...............................................................................................................................................................................   1      1    LT-3
....................ucuggaucuuguucgacagcacu...............................................................................................................................................................................   5      0    JCK-3
....................ucuggaucuuguucgacagcacuc..............................................................................................................................................................................   3      0    JCK-2
```

```
                      Mature                                                                                        Star
uccaucuuguucguuuacccucuggaucuuguucgacagcacucuccuauauuuugccauuuaucuuuaaaugcgaaaucuuuaaaugcaaaauggcgacgaugauaggagugcugucgacaugauccagaaggugcagucgaauaagauacagagggaccacccacaguaccuacucucaacucucaucgcucgcaaucuuauucuucuccucacuuauuauuuuucagaucauuuucuuuuaaucuau
....................ucuggaucuuguucgacagcacuc..............................................................................................................................................................................    5     0    JCK-1
....................ucuggaucuuguucgacagcacuc..............................................................................................................................................................................    3     0    LT-1
....................ucuggaucuuguucgacagcacuU..............................................................................................................................................................................    1     1    LT-1
....................ucuggaucuuguucgacagcacuc..............................................................................................................................................................................    1     0    JCK-3
....................ucuggaucuuguucgacagcacuc..............................................................................................................................................................................    1     0    LT-3
....................ucuggaucuuguucgacagcacucu.............................................................................................................................................................................    1     0    LT-1
....................ucuggaucuuguucgacagcacucu.............................................................................................................................................................................    2     0    JCK-2
.....................cuggaucuuguucgacagc..................................................................................................................................................................................    1     0    LT-1
.....................cuggaucuuguucgacagc..................................................................................................................................................................................    3     0    LT-2
.....................cuggaucuuguucgacagc..................................................................................................................................................................................    1     0    LCK-3
.....................cuggaucuuguucgacagc..................................................................................................................................................................................    4     0    LT-3
.....................cuggaucuuguucgacagca.................................................................................................................................................................................    2     0    LT-1
.....................cuggaucuuguucgacagca.................................................................................................................................................................................    15    0    JCK-1
.....................cuggaucuuguucgacagca.................................................................................................................................................................................    17    0    JCK-2
.....................cuggaucuuguucgacagca.................................................................................................................................................................................    12    0    JCK-3
.....................cuggaucuuguucgacagca.................................................................................................................................................................................    7     0    LCK-3
.....................cuggaucAuguucgacagca.................................................................................................................................................................................    1     1    LT-1
.....................cuggaucuuguucgacagca.................................................................................................................................................................................    4     0    LT-3
.....................cuggaucuuguucgacagcU.................................................................................................................................................................................    1     1    JCK-3
.....................cuggaucuuguucgacagcac................................................................................................................................................................................    2     0    JCK-2
.....................cuggaucuuguucgacagcac................................................................................................................................................................................    4     0    LT-2
.....................cuggaucuuguucgacagcac................................................................................................................................................................................    1     0    JCK-3
.....................cuggaucuuguucgacagcacu...............................................................................................................................................................................    3     0    LT-2
.....................cuggaucuuguucgacagcacu...............................................................................................................................................................................    2     0    LT-3
.....................cuggaucuuguucgacagcacu...............................................................................................................................................................................    27    0    JCK-2
.....................cuggaucuuguucgacagcacu...............................................................................................................................................................................    2     0    LT-1
.....................cuggaucuuguucgacagcacu...............................................................................................................................................................................    5     0    LCK-3
.....................cuggaucuuguucgacagcacu...............................................................................................................................................................................    13    0    JCK-3
.....................cuggaucuuguucgacagcacu...............................................................................................................................................................................    15    0    JCK-1
.....................Uuggaucuuguucgacagcacu...............................................................................................................................................................................    1     1    JCK-3
.....................Uuggaucuuguucgacagcacuc..............................................................................................................................................................................    1     1    JCK-2
.....................cuggaucuuguucgacagcacuc..............................................................................................................................................................................    1     0    JCK-1
.....................cuggaucuuguucgacagcacuc..............................................................................................................................................................................    2     0    LT-3
.....................cuggaucuuguucgacagcacuc..............................................................................................................................................................................    6     0    JCK-2
.....................cuggaucuuguucgacagcacuc..............................................................................................................................................................................    1     0    JCK-3
......................uggaucuuguucgacagU..................................................................................................................................................................................    15    1    LT-3
......................uNgaucuuguucgacagc..................................................................................................................................................................................    1     1    JCK-2
......................uggaucuuguucgacCgc..................................................................................................................................................................................    1     1    LT-2
......................ugNaucuuguucgacagc..................................................................................................................................................................................    4     1    LT-2
......................uggaucuuguucgCcagc..................................................................................................................................................................................    2     1    LCK-2
......................uggaucuuguucgCcagc..................................................................................................................................................................................    1     1    LT-3
......................uggCucuuguucgacagc..................................................................................................................................................................................    19    1    LT-2
......................uggCucuuguucgacagc..................................................................................................................................................................................    6     1    LCK-3
......................uggaucuuguucgaUagc..................................................................................................................................................................................    1     1    LT-2
......................uggaucuuguucgCcagc..................................................................................................................................................................................    3     1    JCK-2
......................uggaucuuguucgaUagc..................................................................................................................................................................................    1     1    LCK-3
......................uggaucuuguucgaAagc..................................................................................................................................................................................    1     1    LCK-3
......................uggaucuuguucgacagc..................................................................................................................................................................................    616   0    JCK-3
......................uggaucuuguucgacGgc..................................................................................................................................................................................    1     1    JCK-2
......................ugAaucuuguucgacagc..................................................................................................................................................................................    1     1    LCK-3
......................uggaucuuguucgacCgc..................................................................................................................................................................................    1     1    JCK-3
......................uAgaucuuguucgacagc..................................................................................................................................................................................    2     1    LCK-3
......................Cggaucuuguucgacagc..................................................................................................................................................................................    1     1    LT-2
......................uggaucuuguucgacagc..................................................................................................................................................................................    454   0    JCK-1
......................uggaucuuguucgacagc..................................................................................................................................................................................    890   0    LT-1
......................ugNaucuuguucgacagc..................................................................................................................................................................................    1     1    LCK-1
......................uggCucuuguucgacagc..................................................................................................................................................................................    1     1    JCK-1
......................uggaucuuguucgCcagc..................................................................................................................................................................................    8     1    LT-2
......................ugUaucuuguucgacagc..................................................................................................................................................................................    1     1    LT-3
......................uggaucuuguucgacagc..................................................................................................................................................................................    1173  0    LT-3
......................ugAaucuuguucgacagc..................................................................................................................................................................................    2     1    JCK-2
......................uggaucuuguucgacaUc..................................................................................................................................................................................    2     1    JCK-3
......................uNgaucuuguucgacagc..................................................................................................................................................................................    1     1    LT-3
......................uggaucuuguucgNcagc..................................................................................................................................................................................    2     1    LCK-1
......................uggaucuuguucgaAagc..................................................................................................................................................................................    1     1    LT-2
......................ugNaucuuguucgacagc..................................................................................................................................................................................    1     1    LCK-3
......................uggaucuuguuNgacagc..................................................................................................................................................................................    1     1    JT-1
......................ugAaucuuguucgacagc..................................................................................................................................................................................    1     1    LCK-1
......................uggaCcuuguucgacagc..................................................................................................................................................................................    4     1    LCK-3
......................uggaucuuguucgCcagc..................................................................................................................................................................................    2     1    JCK-3
```

```
                      Mature                                                                                                   Star
uccaucuuguucguuuacccucuggaucuuguucgacagcacucuccuauauuuugccauuuaucuuuaaaugcgaaaucuuuaaaugcaaaauggcgacgaugauaggagugcugucgacaugauccagaaggugcagucgaauaagauacagagggaccacccacaguaccuacucucaacucucaucgcucgcaaucuuauucuucuccucacuuauuauuuucagaucauuuucuuuuaaucuau
......................uggaucuuguucgacagc.........................................................................................................................................................................................................   663     0     LCK-2
......................uggaucuuguucgacagc.........................................................................................................................................................................................................   794     0     JCK-2
......................uggaucuuguucgacagU.........................................................................................................................................................................................................   5       1     LCK-3
......................uggaucuuguucgacagc.........................................................................................................................................................................................................   74      0     JT-3
......................ugAaucuuguucgacagc.........................................................................................................................................................................................................   2       1     LT-2
......................uggCucuuguucgacagc.........................................................................................................................................................................................................   3       1     LT-1
......................uAgaucuuguucgacagc.........................................................................................................................................................................................................   1       1     LT-2
......................uggCucuuguucgacagc.........................................................................................................................................................................................................   1       1     LCK-1
......................uggaucuuguucgacagc.........................................................................................................................................................................................................   2025    0     LT-2
......................uggGucuuguucgacagc.........................................................................................................................................................................................................   1       1     JT-1
......................uggaucuuguucgacaUc.........................................................................................................................................................................................................   1       1     JCK-1
......................uggaucuuguucgacagU.........................................................................................................................................................................................................   1       1     LT-2
......................uggaucuuguucAacagc.........................................................................................................................................................................................................   1       1     LT-2
......................uggaucuuguucgacagU.........................................................................................................................................................................................................   2       1     JCK-2
......................uggCucuuguucgacagc.........................................................................................................................................................................................................   3       1     LT-3
......................uggaucuuguuNgacagc.........................................................................................................................................................................................................   1       1     JCK-2
......................uggaucuuguucgacagA.........................................................................................................................................................................................................   1       1     LT-3
......................uggaucuuguucgacagc.........................................................................................................................................................................................................   659     0     LCK-1
......................uggaucuuguuNgacagc.........................................................................................................................................................................................................   1       1     JCK-1
......................uggaucuuguucgacaUc.........................................................................................................................................................................................................   6       1     LT-2
......................uggaucuuguucgacagU.........................................................................................................................................................................................................   3       1     JCK-3
......................uggaucuuguucgacaAc.........................................................................................................................................................................................................   1       1     LCK-3
......................uggCucuuguucgacagc.........................................................................................................................................................................................................   3       1     JCK-3
......................uggCucuuguucgacagc.........................................................................................................................................................................................................   1       1     LCK-2
......................uNgaucuuguucgacagc.........................................................................................................................................................................................................   1       1     LCK-1
......................uggaucuuguucUacagc.........................................................................................................................................................................................................   1       1     JCK-3
......................uUgaucuuguucgacagc.........................................................................................................................................................................................................   1       1     LT-3
......................uggaucuuguucgacagU.........................................................................................................................................................................................................   1       1     JCK-1
......................uggaucuuguucgCcagc.........................................................................................................................................................................................................   2       1     LT-1
......................uAgaucuuguucgacagc.........................................................................................................................................................................................................   1       1     LCK-1
......................uggGucuuguucgacagc.........................................................................................................................................................................................................   2       1     JCK-1
......................uggaucuuguucgacaAc.........................................................................................................................................................................................................   1       1     LT-1
......................uggaucuuguucgacagc.........................................................................................................................................................................................................   48      0     JT-2
......................uggaucuuguucgCcagc.........................................................................................................................................................................................................   4       1     LCK-3
......................uggaucuuguucgacagc.........................................................................................................................................................................................................   3342    0     LCK-3
......................uNgaucuuguucgacagc.........................................................................................................................................................................................................   1       1     JT-1
......................uggaucuuguucgCcagc.........................................................................................................................................................................................................   1       1     LCK-1
......................uggaucuuguucgacagU.........................................................................................................................................................................................................   3       1     LT-1
......................uggaucuuguucgaUagc.........................................................................................................................................................................................................   1       1     JCK-1
......................uggaucuuguucgacagc.........................................................................................................................................................................................................   81      0     JT-1
......................ugAaucuuguucgacagc.........................................................................................................................................................................................................   1       1     JCK-3
......................uggaucuuguucgGcagc.........................................................................................................................................................................................................   1       1     LCK-3
......................uggaucuuguucgacaAc.........................................................................................................................................................................................................   1       1     LT-2
......................uAgaucuuguucgacagc.........................................................................................................................................................................................................   2       1     JCK-2
......................uggaucuuguucgCcagca........................................................................................................................................................................................................   2       1     LT-1
......................uggaucuuguucgCcagca........................................................................................................................................................................................................   5       1     LCK-1
......................uggauUuuguucgacagca........................................................................................................................................................................................................   1       1     JCK-3
......................uggaucuuguucgaUagca........................................................................................................................................................................................................   1       1     LT-2
......................Cggaucuuguucgacagca........................................................................................................................................................................................................   1       1     LCK-2
......................uggaucuuguucgaAagca........................................................................................................................................................................................................   1       1     LT-3
......................uggCucuuguucgacagca........................................................................................................................................................................................................   1       1     JCK-3
......................uggaucuuguucgaNagca........................................................................................................................................................................................................   4       1     LCK-1
......................uggaucuuguucgacCgca........................................................................................................................................................................................................   2       1     JCK-2
......................uggaucuuguucNacagca........................................................................................................................................................................................................   1       1     LCK-1
......................uggCucuuguucgacagca........................................................................................................................................................................................................   1       1     LT-3
......................uggaucuuguucgaGagca........................................................................................................................................................................................................   1       1     LCK-1
......................uggaucuuguucgaAagca........................................................................................................................................................................................................   3       1     LCK-2
......................uggaucuuguucgacagcU........................................................................................................................................................................................................   5       1     JT-3
......................uggaucuuguucgacGgca........................................................................................................................................................................................................   1       1     JT-1
......................uggCucuuguucgacagca........................................................................................................................................................................................................   5       1     LT-2
......................uNgaucuuguucgacagca........................................................................................................................................................................................................   3       1     JT-1
......................uggGucuuguucgacagca........................................................................................................................................................................................................   1       1     LCK-3
......................uNgaucuuguucgacagca........................................................................................................................................................................................................   2       1     JCK-3
......................uggaucuuguucgCcagca........................................................................................................................................................................................................   1       1     LCK-2
......................ugNaucuuguucgacagca........................................................................................................................................................................................................   5       1     LT-2
......................uggaucuuguucgacagcC........................................................................................................................................................................................................   1       1     LCK-2
......................uggaucuuguucAacagca........................................................................................................................................................................................................   1       1     LT-3
......................uUgaucuuguucgacagca........................................................................................................................................................................................................   1       1     LT-3
......................uggaucuuguucgaAagca........................................................................................................................................................................................................   1       1     LT-2
......................uggaucuuguucgacaUca........................................................................................................................................................................................................   1       1     LT-3
```

Mature **Star**

uccaucuuguucguuuacccucuggaucuuguucgacagcacucuccuauauuuugccauuuaucuuuaaaugcgaaaucuuuaaaugcaaaauggcgacgaugauaggagugcugucgacaugauccagaaggugcagucgaauaagauacagagggaccacccacaguaccuacucucaacucucaucgcucgcaaucuuauucuucuccucacuuauuauuuuucagaucauuucuuuuaaucuau

uggaucuuguucgaUagca	1	1	JCK-3
uggGucuuguucgacagca	1	1	JT-1
uggaucuuguucgacagca	294	0	JT-3
uggaucuuguucgacagUa	5	1	LT-1
uggaucuuguuNgacagca	5	1	JCK-1
uggaucuuguucgacagca	529	0	JCK-1
uggaucuuguucgacagca	1184	0	LT-2
uggaucuuguucgacaUca	3	1	JCK-2
uggaucuuguuNgacagca	2	1	JT-1
uggaucuuguucgacagUa	4	1	JCK-3
uggaucCuguucgacagca	1	1	JCK-1
uggaucuuguucgacagUa	5	1	LT-3
uggaucuuguucgacagca	1963	0	LCK-2
uAgaucuuguucgacagca	2	1	LCK-3
uggaucuuguucgacagAa	1	1	LT-3
ugCaucuuguucgacagca	1	1	LT-2
uggaucuuAuucgacagca	1	1	LCK-1
uggaucuuguucgacCgca	2	1	LT-2
uNgaucuuguucgacagca	1	1	LCK-1
uggaucuuguucgacagcC	1	1	JT-1
uggaucuuguucgacagUa	1	1	JCK-1
uggCucuuguucgacagca	1	1	JCK-2
uggaucuuguuNgacagca	1	1	JT-2
uggaucuuguucgacagUa	9	1	LCK-3
uggaucuuguucgacagca	1924	0	LCK-1
uggaucuuguucgacagcC	1	1	JCK-2
uggaucuuguucgCcagca	2	1	JCK-3
uggaucuuguucgacaUca	1	1	JCK-3
uggaucuuguucgacagcG	3	1	JCK-2
uggaucuuguucgacagcC	2	1	LT-3
uggaucuuguucgacagca	496	0	LT-1
uggaucuuguucgaUagca	4	1	JCK-1
uggaucuuguucgacagca	606	0	JCK-3
uAgaucuuguucgacagca	1	1	JCK-2
uggCucuuguucgacagca	8	1	LCK-2
uggaucuuguucgacagcU	299	1	LCK-3
uggaucuuguucgacagcG	2	1	LT-3
uggaucCuguucgacagca	1	1	LT-3
uggaucuuguucgacagcU	155	1	JCK-1
uggaucuuguucgacaAca	1	1	JT-1
uAgaucuuguucgacagca	1	1	LT-2
uUgaucuuguucgacagca	1	1	LCK-2
uggaCcuuguucgacagca	2	1	JCK-3
uggaucuuguucgacagcU	56	1	JT-1
uggaucCuguucgacagca	1	1	JT-1
uggaucuuguucgacagUa	11	1	LT-2
uggCucuuguucgacagca	5	1	LT-1
uggaucuuguucgacagcU	75	1	LT-1
uggaucuuguucgacagca	575	0	JT-1
uggCucuuguucgacagca	2	1	LCK-1
uggaucuuguucgacagca	2250	0	LCK-3
uggaucuuguucgCcagca	1	1	JT-1
uNgaucuuguucgacagca	3	1	JCK-2
uggaucuuguucgaUagca	4	1	JCK-2
uggaucuuguucgacagcU	211	1	LT-2
uggaucuuguucgacagcU	162	1	JCK-3
uggNucuuguucgacagca	1	1	JCK-1
uggaucuuguucgacaUca	15	1	LCK-3
Cggaucuuguucgacagca	1	1	LT-2
uggaucuuguucgacagcU	68	1	LCK-1
uggCucuuguucgacagca	2	1	LCK-3
uggaucuuguucgacCgca	1	1	LCK-3
uggGucuuguucgacagca	1	1	JT-2
uggaucuuguucgacagca	932	0	JCK-2
uggaucuuguucgacCgca	1	1	LT-3
uggaucuuguucgacagcU	164	1	LT-3
uggauUuuguucgacagca	1	1	LCK-3
ugAaucuuguucgacagca	1	1	LT-2
uggaucuuguucgacagGa	1	1	JCK-1
uggaucuuguucgacaAca	1	1	LT-1

Mature Star

uccaucuuguucguuuacccucuggaucuuguucgacagcacucuccuauauuuugccauuuaucuuuaaaugcgaaaucuuuaaaugcaaaauggcgacgaugauaggagugcugucgacaugauccagaaggugcagucgaauaagauacagagggaccacccacaguaccuacucucaacucucaucgcucgcaaucuuauucuucuccucacuuauuauuuuucagaucauuuucuuuuaaucuau

......................uggaucuuguucgacagcC......	1	1	LCK-3
......................uggaucuuguucgacaAca......	1	1	LCK-1
......................uggaucuuguucgacagca......	1113	0	LT-3
......................uggaucuuguucgacCgca......	2	1	LCK-2
......................uggaucuuguucgacagGa......	1	1	JCK-2
......................uggaucuuguucgaUagca......	2	1	LCK-2
......................uggauNuuguucgacagca......	3	1	LCK-1
......................uggGucuuguucgacagca......	1	1	LCK-2
......................uggaucuuguucgCcagca......	1	1	LCK-3
......................uggaucuuguucgNcagca......	6	1	LCK-1
......................uggaucuuguucgCcagca......	3	1	LT-2
......................uggaucuuguucgacagcU......	62	1	LCK-2
......................uggaucuuguucgacagUa......	10	1	JT-1
......................uggaucuuguucgacagca......	237	0	JT-2
......................uggCucuuguucgacagca......	1	1	JCK-1
......................ugAaucuuguucgacagca......	1	1	JCK-2
......................uggaucuuguucgacagcU......	194	1	JCK-2
......................uggaucuuguuGgacagca......	9	1	LCK-3
......................uggaucuuguucgacagcU......	9	1	JT-2
......................uggaucCuguucgacagca......	1	1	LCK-1
......................uggaucuuguucgacagUa......	6	1	JCK-2
......................uggCucuuguucgacagcac......	4	1	LCK-2
......................uggaucuuguucgacCgcac......	2	1	JCK-1
......................uggaucuuguucgacagcaU......	19	1	JCK-2
......................uggaucuuguucgCcagcac......	1	1	JCK-1
......................uggCucuuguucgacagcac......	1	1	JT-3
......................uggaucuuguucgacagcCc......	3	1	LT-2
......................uggCucuuguucgacagcac......	2	1	LT-3
......................uggaucuuguucgaGagcac......	1	1	LCK-1
......................uggaCcuuguucgacagcac......	1	1	LCK-3
......................uAgaucuuguucgacagcac......	1	1	LT-3
......................ugUaucuuguucgacagcac......	1	1	JCK-3
......................ugUaucuuguucgacagcac......	1	1	LT-2
......................uggaucuuguucgacagcCc......	1	1	LT-1
......................uggaucuuguucgacagcac......	454	0	JCK-1
......................uggaucuuguucgacagcUc......	2	1	JCK-2
......................uggaucuuguucgacagcac......	762	0	LT-3
......................uggaucuuguucgaAagcac......	1	1	JCK-1
......................uggaucuuguucgacagcGc......	1	1	LT-2
......................uggaucuuguucgaAagcac......	1	1	LT-3
......................uggaucuuguucgGcagcac......	1	1	JCK-1
......................uggaucuuguucgacagcUc......	1	1	LT-1
......................uggaucuuguucgacCgcac......	1	1	LT-2
......................uggaucuuguucgacagcac......	796	0	LCK-2
......................ugAaucuuguucgacagcac......	2	1	JCK-1
......................uggaucuuguucgaNagcac......	4	1	LCK-1
......................uggaucuuguucgacagUac......	1	1	JCK-3
......................uggaucuuguucgacagcUc......	3	1	LT-3
......................uggaucuuguucgaUagcac......	1	1	LT-2
......................uggaucuuguucgacagcaA......	9	1	JCK-3
......................uggaucuuguucgacagcaA......	1	1	JCK-1
......................uggaucuuguucUacagcac......	1	1	LT-1
......................uggaucuuguucgCcagcac......	1	1	LT-3
......................uggaucuuguucgacagcaA......	3	1	JCK-2
......................uggCucuuguucgacagcac......	2	1	JCK-2
......................uggaucuuguucgacagcNc......	1	1	LCK-1
......................uggaucuuguucgacagcaU......	10	1	LT-1
......................uggaucuuguucgacagcac......	564	0	LT-1
......................uggCucuuguucgacagcac......	1	1	LT-1
......................uggaucuuguucgacagcUc......	1	1	JCK-1
......................uggaucuuguucgGcagcac......	1	1	LCK-1
......................uggaucuuguucgacaAcac......	1	1	JCK-1
......................uggaucuuguucgacagcac......	727	0	JCK-2
......................uggaucuuguucgacagcaU......	5	1	JCK-1
......................uggaucuuguucgacGgcac......	1	1	LT-1
......................uggaucuuguucgacagcCc......	1	1	LCK-2
......................ugUaucuuguucgacagcac......	1	1	LT-3
......................Cggaucuuguucgacagcac......	2	1	LT-2
......................uggaucuuguuNgacagcac......	1	1	JT-2
......................uggaucuuguucgCcagcac......	6	1	LCK-3

Mature **Star**

uccaucuuguucguuuacccucuggaucuuguucgacagcacucuccuauauuuugccauuuaucuuuaaaugcgaaaucuuuaaaugcaaaauggcgacgaugauaggagugcugucgacaugauccagaaggugcagucgaauaagauacagagggaccacccacaguaccuacucucaacucucaucgcucgcaaucuuauucuucuccucacuuauuauuuucagaucauuucuuuuaaucuau			
......................uggaucuuguucAacagcac	1	1	LT-2
......................uggaucuuguucgacagcac	508	0	JCK-3
......................uggaucuuguucgacGgcac	1	1	JCK-2
......................uggaucuuguucgacagcCc	1	1	LCK-3
......................uggaucuuguucgaUagcac	1	1	JCK-1
......................uNgaucuuguucgacagcac	1	1	JCK-1
......................uggaucuugCucgacagcac	1	1	LT-2
......................uggaucuuguucgCcagcac	5	1	LT-2
......................uggaucuuguucgacagcaU	12	1	JCK-3
......................uggCucuuguucgacagcac	5	1	LCK-3
......................uggaucuuguucgNcagcac	4	1	LCK-1
......................uggaucuuguucgacagcaU	16	1	LT-3
......................uggaucuuguucgacagcCc	1	1	JT-3
......................uggaucuuguucgacagcUc	2	1	JCK-3
......................uggauNuuguucgacagcac	2	1	LCK-1
......................uggaucuuguucgacGgcac	1	1	LT-2
......................uggaucuuguucgacagcac	164	0	JT-1
......................uggaucuuguucgacagcac	72	0	JT-2
......................uggaucuuguucgacagcac	821	0	LCK-1
......................uggaucuuguucgacagcac	1028	0	LT-2
......................uggCucuuguucgacagcac	11	1	LT-2
......................ugAaucuuguucgacagcac	1	1	LCK-3
......................uggaucuuguucgacagcUc	3	1	LT-2
......................uggaucuuguucgaUagcac	3	1	JCK-2
......................uggaucuuguucgacagcNc	1	1	JCK-1
......................uggaucuuguucgaUagcac	3	1	JCK-3
......................uggaucuuguucgCcagcac	1	1	LCK-2
......................uggaucuuguucgacagcaU	1	1	LCK-3
......................uggCucuuguucgacagcac	2	1	LCK-1
......................ugAaucuuguucgacagcac	1	1	LT-2
......................uggaucuuguucgacagcaA	2	1	LT-3
......................uggaucuuguucgaAagcac	2	1	LCK-2
......................uggaucuuAuucgacagcac	1	1	LCK-1
......................uggaucuuguucgacagcac	2109	0	LCK-3
......................uAgaucuuguucgacagcac	1	1	LCK-3
......................uggaucuuguucgacagcac	83	0	JT-3
......................ugNaucuuguucgacagcac	4	1	LT-2
......................uggaucuuguucgacagcaU	11	1	LT-2
......................uggaucAuguucgacagcacu	1	1	JCK-2
......................uggaucCuguucgacagcacu	1	1	JT-1
......................uggaucuuguucgacagcaUu	17	1	JCK-3
......................uggaucuuguucgacagcacC	1	1	LT-1
......................uggaucCuguucgacagcacu	2	1	JCK-1
......................ugAaucuuguucgacagcacu	8	1	JCK-2
......................uggaucuuguucgacagcacu	1125	0	JT-1
......................uggaucuuguuGgacagcacu	10	1	JCK-1
......................uggaucCuguucgacagcacu	2	1	LCK-3
......................uAgaucuuguucgacagcacu	2	1	LCK-2
......................Cggaucuuguucgacagcacu	1	1	LCK-3
......................uggaucuuguucgCcagcacu	16	1	LT-2
......................uggaucuuguucgacagcacA	37	1	JT-1
......................uggaucuuguucgacagcacA	24	1	LT-3
......................uggaucuCguucgacagcacu	1	1	LCK-2
......................uggaucuuguucgacagcGcu	2	1	JCK-3
......................uggaucuuguucgacagcacC	22	1	JCK-2
......................uggaucuuguucgacagcacC	5	1	JCK-1
......................uAgaucuuguucgacagcacu	7	1	LCK-3
......................uggaucuuguuNgacagcacu	121	1	JCK-1
......................uggGucuuguucgacagcacu	9	1	JCK-1
......................ugAaucuuguucgacagcacu	2	1	LCK-3
......................uggCucuuguucgacagcacu	55	1	LT-2
......................uggaucuuguucgacaAcacu	1	1	LCK-3
......................uggaucuuguucgacagUacu	23	1	JCK-2
......................uggaucuuguucgCcagcacu	10	1	LT-3
......................uggaucCuguucgacagcacu	5	1	JCK-3
......................uggaucuuguucgacaAcacu	2	1	JCK-3
......................uggaucCuguucgacagcacu	2	1	LT-2
......................uNgaucuuguucgacagcacu	29	1	JCK-3
......................uggaucuugCucgacagcacu	3	1	JCK-3
......................uggaucuuguucgacagAacu	1	1	JCK-2

Mature **Star**

uccaucuuguucguuuacccucuggaucuuguucgacagcacucuccuauauuuugccauuuaucuuuaaaugcgaaaucuuuaaaugcaaaauggcgacgaugauaggagugcugucgacaugauccagaaggugcagucgaauaagauacagagggaccacccacaguaccuacucucaacucucaucgcucgcaaucuuauucuucuccucacuuauuauuuucagaucauuucuuuuaaucuau

uAgaucuuguucgacagcacu	3	1	LT-3
uggaucuuguucgacagcCcu	2	1	LCK-3
uggaucuuguucgacagNacu	3	1	JCK-3
uggaucuuguCcgacagcacu	2	1	JCK-2
uggaucuuguucgacGgcacu	1	1	LCK-2
uggaucuuguucgaNagcacu	4	1	LCK-1
uAgaucuuguucgacagcacu	10	1	JCK-2
uggaucuuguucgacGgcacu	1	1	LCK-3
uggaucuuguucgacagcUcu	2	1	LT-1
ugAaucuuguucgacagcacu	11	1	JCK-3
uggaucuuguucgacagcaUu	7	1	JT-1
uggCucuuguucgacagcacu	1	1	JT-2
uggaucuuguucgacagcGcu	7	1	JCK-2
uggaucuuguCcgacagcacu	2	1	JCK-1
uggaucuuguuNgacagcacu	1	1	JT-1
uAgaucuuguucgacagcacu	3	1	LT-2
uggauNuuguucgacagcacu	1	1	JCK-1
uggaucuuguucgacagcaAu	1	1	LCK-3
uggaucuuguucgCcagcacu	47	1	JCK-1
uggaucuuguucgacagcacA	79	1	JCK-2
uggaCcuuguucgacagcacu	1	1	LCK-3
uggaucuuguucUacagcacu	4	1	JCK-2
uggaucuuguucgacagcacC	3	1	LT-3
uggaucuuguucUacagcacu	1	1	JCK-1
uggaucuuguucgacagcacu	1264	0	LCK-1
uggCucuuguucgacagcacu	75	1	JCK-3
uggaucuuguucgacagcacu	5544	0	LCK-3
uggaucuuguucgacaAcacu	8	1	JCK-1
uggaucuuguucgacagcaAu	2	1	LT-3
uggaucuCguucgacagcacu	1	1	JCK-1
uggaucuuguucgacagcacG	6	1	LCK-3
uAgaucuuguucgacagcacu	1	1	LT-1
uCgaucuuguucgacagcacu	1	1	JCK-3
uggaucuuguucgNcagcacu	5	1	LCK-1
uggaucuuguucgacagcacu	4196	0	LT-2
uggaucuuguucgacagcacu	277	0	JT-2
uggaucuuguucgaGagcacu	1	1	JCK-3
ugUaucuuguucgacagcacu	1	1	LCK-3
uggauUuuguucgacagcacu	1	1	JCK-1
uggaucuuguucgacagcaAu	12	1	JCK-1
uggaucuuguucgacagcaUu	38	1	LCK-3
uggaucuuguucgacagcacu	1314	0	LCK-2
ugCaucuuguucgacagcacu	1	1	JCK-1
uggaucuuguucgacagcacA	51	1	JCK-1
uggaucuuCuucgacagcacu	1	1	JCK-3
uggaucuuAuucgacagcacu	1	1	LT-3
uggaucuuguuAgacagcacu	1	1	LCK-3
uggaucuuguucgacagcacC	4	1	JT-3
uggauUuuguucgacagcacu	4	1	JCK-2
uggaucuuguucgacagcacC	8	1	LT-2
uggaucuuAuucgacagcacu	1	1	JCK-3
uggaucuuguucgacGgcacu	2	1	JCK-1
uggaucuuguuGgacagcacu	1	1	JCK-2
uggaucuuUuucgacagcacu	3	1	JCK-2
uggaucuuguucgacaUcacu	2	1	JCK-1
uggaucuuguucgacagcaUu	13	1	JCK-1
uUgaucuuguucgacagcacu	3	1	JCK-2
uggaucuuguucgacagcUcu	1	1	JCK-2
uggaucuuguucgaUagcacu	26	1	JCK-2
uggaucuuguucgaUagcacu	2	1	LT-1
ugNaucuuguucgacagcacu	2	1	LCK-1
uggaucuuguucgacGgcacu	1	1	LT-2
uggaucuuguucgaUagcacu	11	1	JCK-3
uggaucuuguucgaUagcacu	2	1	LT-3
ugAaucuuguucgacagcacu	6	1	JCK-1
ugAaucuuguucgacagcacu	2	1	LT-2
uggaucuuguucgaUagcacu	12	1	JCK-1
uggNucuuguucgacagcacu	1	1	LCK-1
Cggaucuuguucgacagcacu	2	1	JCK-3
Gggaucuuguucgacagcacu	1	1	JCK-3

```
                      Mature                                                                                        Star
uccaucuuguucguuuacccucuggaucuuguucgacagcacucuccuauauuuugccauuuaucuuuaaaugcgaaaucuuuaaaugcaaaauggcgacgaugauaggagugcugucgacaugauccagaaggugcagucgaauaagauacagagggaccacccacaguaccuacucucaacucucaucgcucgcaaucuuauucuucuccucacuuauuauuuucagaucauuucuuuuaaucuau
......................uggaucuCguucgacagcacu.............................................................................................................................................................................................................   1    1    LT-1
......................uggaucuuguucgacagcaUu.............................................................................................................................................................................................................   18   1    LT-1
......................uggaucuuguucgacagcacA.............................................................................................................................................................................................................   50   1    LT-2
......................uggaCcuuguucgacagcacu.............................................................................................................................................................................................................   4    1    JCK-3
......................uggaucuuguucgacaAcacu.............................................................................................................................................................................................................   9    1    JCK-2
......................uCgaucuuguucgacagcacu.............................................................................................................................................................................................................   3    1    JCK-2
......................uggaucuuguucgacagcCcu.............................................................................................................................................................................................................   1    1    LCK-2
......................uggaucuuguucgacagcacG.............................................................................................................................................................................................................   2    1    LT-3
......................uggCucuuguucgacagcacu.............................................................................................................................................................................................................   19   1    LT-3
......................uggaucuuguucgaAagcacu.............................................................................................................................................................................................................   1    1    JCK-1
......................uggaCcuuguucgacagcacu.............................................................................................................................................................................................................   5    1    JCK-2
......................uggaucuuguucgacagcCcu.............................................................................................................................................................................................................   24   1    JCK-2
......................uggaucuuguucgacagcCcu.............................................................................................................................................................................................................   7    1    LT-2
......................uggaucuuguucgacagcaAu.............................................................................................................................................................................................................   3    1    LT-1
......................uggaucuuguucgacagGacu.............................................................................................................................................................................................................   7    1    JCK-1
......................uggaucuuguucgacagcaAu.............................................................................................................................................................................................................   1    1    LCK-2
......................uggaucuuguucgacaCcacu.............................................................................................................................................................................................................   1    1    JCK-1
......................uggaucuuguCcgacagcacu.............................................................................................................................................................................................................   1    1    LT-3
......................uggaucuuguucgacagcacA.............................................................................................................................................................................................................   16   1    LT-1
......................uggaucuuguucgaAagcacu.............................................................................................................................................................................................................   9    1    JCK-3
......................uggaucuuguucgacagcCcu.............................................................................................................................................................................................................   17   1    JCK-3
......................uggaucuuguucgacCgcacu.............................................................................................................................................................................................................   13   1    JCK-2
......................uggaucuuguucgacaAcacu.............................................................................................................................................................................................................   1    1    LT-1
......................uggaucuuguucAacagcacu.............................................................................................................................................................................................................   6    1    JCK-1
......................uggCucuuguucgacagcacu.............................................................................................................................................................................................................   67   1    JCK-1
......................uggauUuuguucgacagcacu.............................................................................................................................................................................................................   4    1    JCK-3
......................uggaucuuguucgacagUacu.............................................................................................................................................................................................................   19   1    JCK-3
......................uggaucuuguucgacagcaGu.............................................................................................................................................................................................................   1    1    JCK-1
......................uggCucuuguucgacagcacu.............................................................................................................................................................................................................   18   1    LCK-3
......................uggaucuuguucgacGgcacu.............................................................................................................................................................................................................   5    1    JCK-2
......................uggaucuuguucgGcagcacu.............................................................................................................................................................................................................   2    1    LCK-1
......................uggGucuuguucgacagcacu.............................................................................................................................................................................................................   1    1    LCK-3
......................uggaucuCguucgacagcacu.............................................................................................................................................................................................................   2    1    JCK-3
......................uNgaucuuguucgacagcacu.............................................................................................................................................................................................................   2    1    LT-3
......................uggaucuuguucgacagcGcu.............................................................................................................................................................................................................   1    1    LT-3
......................uggaucuuguucgacagcaNu.............................................................................................................................................................................................................   23   1    JCK-1
......................uggaucuuguucgacCgcacu.............................................................................................................................................................................................................   1    1    LCK-2
......................uggaucuuAuucgacagcacu.............................................................................................................................................................................................................   2    1    JCK-1
......................uggaucuuguucgacaUcacu.............................................................................................................................................................................................................   4    1    JCK-3
......................uggaucuuguuAgacagcacu.............................................................................................................................................................................................................   1    1    JCK-2
......................Cggaucuuguucgacagcacu.............................................................................................................................................................................................................   2    1    LT-3
......................uggaucuuguucgacagcUcu.............................................................................................................................................................................................................   1    1    JCK-3
......................uggaucuuguucgacagcacN.............................................................................................................................................................................................................   6    1    JCK-1
......................uggaucuuguucgGcagcacu.............................................................................................................................................................................................................   2    1    JCK-3
......................uggaucuuUuucgacagcacu.............................................................................................................................................................................................................   3    1    JCK-3
......................uggaucuuguucgacagcacC.............................................................................................................................................................................................................   2    1    JT-2
......................uggaucuuguucgCcagcacu.............................................................................................................................................................................................................   6    1    LCK-3
......................uggaucuuAuucgacagcacu.............................................................................................................................................................................................................   10   1    JCK-2
......................uggaucuuguucgacagcacC.............................................................................................................................................................................................................   2    1    LCK-3
......................uggaucuuguucgCcagcacu.............................................................................................................................................................................................................   80   1    JCK-2
......................uNgaucuuguucgacagcacu.............................................................................................................................................................................................................   37   1    JCK-2
......................uUgaucuuguucgacagcacu.............................................................................................................................................................................................................   1    1    JCK-1
......................uggaucuuguucgacagcaAu.............................................................................................................................................................................................................   3    1    LT-2
......................uggaucuuguucgacagcaUu.............................................................................................................................................................................................................   17   1    LT-3
......................uggaucuuguucgCcagcacu.............................................................................................................................................................................................................   2    1    JT-3
......................uggaucuuguucgacagcaUu.............................................................................................................................................................................................................   17   1    LT-2
......................ugAaucuuguucgacagcacu.............................................................................................................................................................................................................   2    1    LT-1
......................uggaucuuguucgacaUcacu.............................................................................................................................................................................................................   5    1    JCK-2
......................uggaucCuguucgacagcacu.............................................................................................................................................................................................................   7    1    JCK-2
......................uggaucuuguuGgacagcacu.............................................................................................................................................................................................................   1    1    JCK-3
......................uggaucuuguuGgacagcacu.............................................................................................................................................................................................................   1    1    LT-3
......................uggaucuuguucgacagcCcu.............................................................................................................................................................................................................   1    1    LCK-1
......................uUgaucuuguucgacagcacu.............................................................................................................................................................................................................   6    1    JCK-3
......................uggaucuuguucgacGgcacu.............................................................................................................................................................................................................   1    1    LT-1
......................uggCucuuguucgacagcacu.............................................................................................................................................................................................................   4    1    JT-1
......................uUgaucuuguucgacagcacu.............................................................................................................................................................................................................   2    1    LCK-3
......................uggaucuuguucgacaUcacu.............................................................................................................................................................................................................   1    1    LT-2
......................uggaucGuguucgacagcacu.............................................................................................................................................................................................................   1    1    JCK-3
......................uggGucuuguucgacagcacu.............................................................................................................................................................................................................   3    1    JCK-2
......................uggaucuCguucgacagcacu.............................................................................................................................................................................................................   7    1    JCK-2
```

Mature   Star

uccaucuuguucguuuacccucuggaucuuguucgacagcacucuccuauauuuugccauuuaucuuuaaaugcgaaaucuuuaaaugcaaaauggcgacgaugauaggagugcugucgacaugauccagaaggugcagucgaauaagauacagagggaccacccacaguaccuacucucaacucucaucgcucgcaaucuuauucuucuccucacuuauuuuucagaucauuuucuuuuaaucuau

uggaucuuguucgCcagcacu	2	1	LCK-2
uggNucuuguucgacagcacu	21	1	JCK-1
uggaucuuguucgacCgcacu	7	1	JCK-1
uCgaucuuguucgacagcacu	1	1	LT-2
uggaucuuguucgacagcaUu	38	1	JCK-2
uggaucuuguucgacagcacA	61	1	LCK-3
Aggaucuuguucgacagcacu	1	1	JCK-2
uggaucuuguucgacagcacG	2	1	LT-1
uggCucuuguucgacagcacu	104	1	JCK-2
uggCucuuguucgacagcacu	1	1	LCK-1
uggCucuuguucgacagcacu	10	1	LT-1
Cggaucuuguucgacagcacu	2	1	JCK-1
uggaucuuguucgacagcGcu	1	1	JCK-1
uggaucuuguucAacagcacu	7	1	JCK-2
uggaucuuguucgacagcacA	59	1	JCK-3
ugUaucuuguucgacagcacu	1	1	LT-1
uggaucuuguucgacagcacu	26265	0	JCK-3
uAgaucuuguucgacagcacu	9	1	JCK-1
uggaucuuguucgacagcCcu	1	1	JT-1
uggaucuuguucgacagcacu	35956	0	JCK-2
uggaucuuguucCacagcacu	1	1	JCK-2
uggaucuuguucUacagcacu	6	1	JCK-3
uggaucuuguucNacagcacu	1	1	LCK-1
uAgaucuuguucgacagcacu	1	1	JT-1
uggaucuuguucgacagcCcu	2	1	LT-3
Cggaucuuguucgacagcacu	8	1	JCK-2
uggaucuuguucgacagcacu	20810	0	JCK-1
uggaucuuguucgCcagcacu	48	1	JCK-3
uggaucuuguucgacagcacu	4453	0	LT-3
uggaCcuuguucgacagcacu	1	1	JCK-1
uggaCcuuguucgacagcacu	1	1	LCK-2
ugNaucuuguucgacagcacu	1	1	LT-1
uggNucuuguucgacagcacu	1	1	JCK-2
ugUaucuuguucgacagcacu	2	1	JCK-1
uggaucuuguucgaAagcacu	5	1	JCK-2
uggGucuuguucgacagcacu	4	1	JCK-3
uggaucuuguucgacagUacu	1	1	LT-1
uggaucuuguucgacagcacG	12	1	JCK-1
uggNucuuguucgacagcacu	4	1	JCK-3
uggCucuuguucgacagcacu	2	1	LCK-2
uggaucuuAuucgacagcacu	3	1	LCK-3
uggaucuuguucgacagcaAu	2	1	JCK-3
Cggaucuuguucgacagcacu	2	1	LT-2
uggaucuuNuucgacagcacu	2	1	JCK-3
uggaucuuguucgacagcacu	2933	0	LT-1
uggaucuuguucgCcagcacu	8	1	LT-1
uggaucuuAuucgacagcacu	1	1	LT-2
uggaucuuguucgCcagcacu	2	1	JT-1
uggaucuuguuAgacagcacu	1	1	JCK-1
uggauNuuguucgacagcacu	1	1	LCK-1
uggaucuuguucgacagcaAu	3	1	JCK-2
ugAaucuuguucgacagcacu	1	1	LT-3
uggaucuuguucAacagcacu	1	1	JCK-3
uggaucCuguucgacagcacu	1	1	JT-3
uggaucuugCucgacagcacu	9	1	JCK-2
ugUaucuuguucgacagcacu	7	1	JCK-3
uggaucuuguucgacagcacC	10	1	JCK-3
uggaucuuguucgacagcacG	15	1	JCK-2
ugNaucuuguucgacagcacu	9	1	LT-2
uggaucuuguCcgacagcacu	1	1	JCK-3
uggaucuGguucgacagcacu	6	1	JCK-2
uggaucuuguucgGcagcacu	2	1	JCK-1
uggaucuugCucgacagcacu	3	1	JCK-1
uAgaucuuguucgacagcacu	7	1	JCK-3
uggaucuuguucgacagcCcu	11	1	JCK-1
uggaucuuguucgacagcacG	12	1	JCK-3
uggaucuuguucgacagcacu	423	0	JT-3
uggaucuuguucgacCgcacu	10	1	JCK-3
uggaucuuguucgacagcCcu	3	1	LT-1
Aggaucuuguucgacagcacu	4	1	LT-2

Mature **Star**

uccaucuuguucguuuacccucuggaucuuguucgacagcacucuccuauauuuugccauuuaucuuuaaaugcgaaaucuuuaaaugcaaaauggcgacgaugauaggagugcugucgacaugauccagaaggugcagucgaauaagauacagagggaccacccacaguaccuacucucaacucucaucgcucgcaaucuuauucuucuccucacuuauuauuuucagaucauuuucuuuuaaucuau			
......................uUgaucuuguucgacagcacu	1	1	LT-2
......................uNgaucuuguucgacagcacu	31	1	JCK-1
......................uggaucuuguucgacagcNcu	10	1	JCK-1
......................uggaucuuguucgacCgcacu	3	1	LT-3
......................uggaucuuguucAacagcacu	1	1	LCK-2
......................uggaucuuguucgacGgcacu	10	1	JCK-3
......................uggaucuuguucCacagcacu	4	1	LCK-3
......................uggaucuuguGcgacagcacu	3	1	JCK-1
......................uggaucuuguucgacagUacu	16	1	JCK-1
......................uggaucuugCucgacagcacu	6	1	LCK-3
......................ugUaucuuguucgacagcacu	3	1	JCK-2
......................uggaucuuguucgacCgcacu	3	1	LT-2
......................uggaucuuguucAacagcacu	1	1	LCK-1
......................uggaucuuguuNgacagcacu	1	1	JCK-2
......................uggaucuuguucgacagcacAc	1	1	LT-1
......................uggCucuuguucgacagcacuc	7	1	LT-3
......................uggaucuuguucgCcagcacuc	17	1	JCK-1
......................uggaucuuguucgacagcacuA	11	1	LCK-2
......................uggaucuuguucgGcagcacuc	1	1	LCK-1
......................uNgaucuuguucgacagcacuc	1	1	LT-3
......................uggGucuuguucgacagcacuc	4	1	JCK-1
......................uggaucuuguGcgacagcacuc	5	1	JCK-1
......................uggaucuuguucgacGgcacuc	4	1	JCK-3
......................uAgaucuuguucgacagcacuc	3	1	JCK-1
......................uggaucuuguucgacagNacuc	1	1	JCK-3
......................uggaucuuguucgUcagcacuc	1	1	JCK-1
......................uggaucuuguucUacagcacuc	2	1	LT-1
......................uggaucuuguucgacagAacuc	1	1	JCK-1
......................uggaucuuguucgacagcacuU	1	1	LCK-1
......................uggaucuuguucgacagcCcuc	9	1	JCK-2
......................uUgaucuuguucgacagcacuc	2	1	JCK-3
......................uggaucuuguucgacagcGcuc	1	1	JCK-3
......................uggaucuuguucgacagcacuc	58	0	JT-2
......................uggaucuuNuucgacagcacuc	1	1	JCK-3
......................uggaucuuguucgacagcacuA	12	1	LT-2
......................uggaucuuguucgacagcacuc	2355	0	LT-3
......................uggaCcuuguucgacagcacuc	2	1	JCK-2
......................uggNucuuguucgacagcacuc	8	1	JCK-1
......................uggNucuuguucgacagcacuc	1	1	JCK-3
......................uggGucuuguucgacagcacuc	1	1	LT-3
......................uggaucuuguucgacagcacuG	2	1	JCK-3
......................uggaucCuguucgacagcacuc	2	1	LCK-3
......................uggaucuuguucgacagcacuc	10812	0	JCK-3
......................uggaucuuguucgacagGacuc	6	1	JCK-1
......................uggaucuuguucgacagcacuG	12	1	JCK-2
......................uggaucuuguucAacagcacuc	1	1	LT-2
......................uggaucuuguucgacagcaUuc	1	1	LT-1
......................uggaucuuguucgacagcacuc	2023	0	LT-1
......................uggaucuuguucgaAagcacuc	5	1	JCK-2
......................uggaucuuguucgacagcacuU	31	1	LCK-3
......................uggaucuuguucgacagcacuU	81	1	JCK-3
......................uggaucuuAuucgacagcacuc	2	1	JCK-2
......................uggaucuuguucgacagcacuc	84	0	LCK-2
......................uggaucuuguucgacCgcacuc	1	1	LCK-2
......................uggaucuuguucgacagcacCc	1	1	LT-3
......................uggaucuuAuucgacagcacuc	1	1	JCK-1
......................uggaucuuguCcgacagcacuc	1	1	JCK-3
......................uggaucuuguucgacagcacuA	14	1	LCK-1
......................uggaucCuguucgacagcacuc	1	1	LT-3
......................uggaucuuguucgacagcNcuc	3	1	JCK-1
......................uggaucuuguucgacagcacuc	955	0	LCK-3
......................ugAaucuuguucgacagcacuc	5	1	JCK-3
......................uggaucuuguucgacagUacuc	4	1	JCK-1
......................uggaucuuguucgacagcacuA	36	1	LCK-3
......................uAgaucuuguucgacagcacuc	2	1	LT-2
......................uggaucuuguucgacagcacCc	2	1	JCK-3
......................uggaucuuguuGgacagcacuc	3	1	JCK-1
......................uggaucuuguucAacagcacuc	2	1	JCK-3
......................ugNaucuuguucgacagcacuc	2	1	LT-2
......................Cggaucuuguucgacagcacuc	3	1	JCK-2

```
                      Mature                                                                                        Star
uccaucuuguucguuuacccucuggaucuuguucgacagcacucuccuauauuuugccauuuaucuuuaaaugcgaaaucuuuaaaugcaaaauggcgacgaugauaggagugcugucgacaugauccagaaggugcagucgaauaagauacagagggaccacccacaguaccuacucucaacucucaucgcucgcaaucuuauucuucuccucacuuauuauuuucagaucauuuucuuuuaaucuau
......................uggaucuuguucgacCgcacuc.............................................................................................................................................................................................................    1      1    LT-3
......................uggaucuuguucgacagcacuA.............................................................................................................................................................................................................    19     1    LT-1
......................uggaucuuguucgacagcCcuc.............................................................................................................................................................................................................    2      1    LT-1
......................uggaCcuuguucgacagcacuc.............................................................................................................................................................................................................    3      1    JCK-3
......................uggauUuuguucgacagcacuc.............................................................................................................................................................................................................    2      1    JCK-3
......................uggaucuuguucgacagcacuc.............................................................................................................................................................................................................    14605  0    JCK-2
......................uggaucuuguucUacagcacuc.............................................................................................................................................................................................................    2      1    JCK-1
......................uggaucuuguucgacGgcacuc.............................................................................................................................................................................................................    2      1    JCK-2
......................uggCucuuguucgacagcacuc.............................................................................................................................................................................................................    24     1    JCK-3
......................uggaucuuguucgacGgcacuc.............................................................................................................................................................................................................    1      1    JCK-1
......................uggaucuuguucgaAagcacuc.............................................................................................................................................................................................................    3      1    JCK-3
......................uggaucuuguucgacagcacuG.............................................................................................................................................................................................................    3      1    LT-1
......................uggaucuuguucgacagcacuA.............................................................................................................................................................................................................    47     1    JCK-1
......................uNgaucuuguucgacagcacuc.............................................................................................................................................................................................................    15     1    JCK-2
......................uggaucuuguucgacCgcacuc.............................................................................................................................................................................................................    8      1    JCK-2
......................uggaucuuguucgacagcCcuc.............................................................................................................................................................................................................    3      1    JCK-1
......................uggaucuuguucgacagcaNuc.............................................................................................................................................................................................................    5      1    JCK-1
......................uggaucCuguucgacagcacuc.............................................................................................................................................................................................................    1      1    JCK-2
......................uggaucuuguucgaUagcacuc.............................................................................................................................................................................................................    2      1    LT-1
......................uggaucuuguucgacagcaUuc.............................................................................................................................................................................................................    14     1    JCK-2
......................uggaucuuguucgacaUcacuc.............................................................................................................................................................................................................    1      1    JCK-1
......................uggaucuuguucgacagcacAc.............................................................................................................................................................................................................    1      1    JCK-2
......................uggaucuuguucUacagcacuc.............................................................................................................................................................................................................    1      1    JCK-2
......................uggaucuuguucgacagcGcuc.............................................................................................................................................................................................................    2      1    JCK-1
......................uggaucuCguucgacagcacuc.............................................................................................................................................................................................................    1      1    JCK-1
......................uggaucuuguucgCcagcacuc.............................................................................................................................................................................................................    5      1    LT-3
......................ugAaucuuguucgacagcacuc.............................................................................................................................................................................................................    1      1    LT-1
......................uggaucuuguucCacagcacuc.............................................................................................................................................................................................................    5      1    JCK-2
......................uNgaucuuguucgacagcacuc.............................................................................................................................................................................................................    11     1    JCK-1
......................uggaucuuguucgacagcacuc.............................................................................................................................................................................................................    8479   0    JCK-1
......................uggaucuuguucgacagcacNc.............................................................................................................................................................................................................    4      1    JCK-1
......................uggCucuuguucgacagcacuc.............................................................................................................................................................................................................    3      1    LT-1
......................uggaucuuguucgacagcacuc.............................................................................................................................................................................................................    74     0    LCK-1
......................uggaucuCguucgacagcacuc.............................................................................................................................................................................................................    1      1    JCK-3
......................uggaucuuguucgaUagcacuc.............................................................................................................................................................................................................    26     1    JCK-3
......................uggCucuuguucgacagcacuc.............................................................................................................................................................................................................    43     1    JCK-2
......................uggaucuCguucgacagcacuc.............................................................................................................................................................................................................    1      1    LCK-3
......................uggaucuuguucgacaUcacuc.............................................................................................................................................................................................................    1      1    JCK-3
......................uggaucuuguucgacagUacuc.............................................................................................................................................................................................................    3      1    JCK-3
......................uggaucuuguucgacaAcacuc.............................................................................................................................................................................................................    1      1    LT-1
......................uAgaucuuguucgacagcacuc.............................................................................................................................................................................................................    4      1    JCK-2
......................uggaCcuuguucgacagcacuc.............................................................................................................................................................................................................    1      1    JCK-1
......................uggaucuuguucgacagcaUuc.............................................................................................................................................................................................................    7      1    JCK-1
......................uggaucuuguucgaUagcacuc.............................................................................................................................................................................................................    10     1    JCK-1
......................uggaucuuguucgacagcacuc.............................................................................................................................................................................................................    188    0    JT-1
......................uggaucuuguucgacagUacuc.............................................................................................................................................................................................................    5      1    JCK-2
......................uggCucuuguucgacagcacuc.............................................................................................................................................................................................................    8      1    LT-2
......................uggaucuuguucCacagcacuc.............................................................................................................................................................................................................    1      1    JCK-3
......................uggaucuuguucgacagcacuG.............................................................................................................................................................................................................    9      1    JCK-1
......................uggaucuuguucgacagcacuA.............................................................................................................................................................................................................    9      1    JT-1
......................uggaucuugCucgacagcacuc.............................................................................................................................................................................................................    5      1    JCK-1
......................uNgaucuuguucgacagcacuc.............................................................................................................................................................................................................    1      1    LCK-3
......................uggaucuuguucCacagcacuc.............................................................................................................................................................................................................    2      1    JCK-1
......................uggaucuuguucgacagcacuA.............................................................................................................................................................................................................    24     1    LT-3
......................uggaucuugCucgacagcacuc.............................................................................................................................................................................................................    2      1    LT-1
......................uggaucuuguucgacagcaAuc.............................................................................................................................................................................................................    1      1    JCK-1
......................uggaucuuAuucgacagcacuc.............................................................................................................................................................................................................    1      1    LT-3
......................uggaucuuguucgacaAcacuc.............................................................................................................................................................................................................    4      1    JCK-2
......................uggaucuuguucgacagcacuG.............................................................................................................................................................................................................    1      1    LT-3
......................uggaucuuguucgacagcacGc.............................................................................................................................................................................................................    1      1    JCK-1
......................uggaucuuguucgUcagcacuc.............................................................................................................................................................................................................    1      1    JCK-3
......................uggaucuuUuucgacagcacuc.............................................................................................................................................................................................................    1      1    JCK-1
......................uggaucCuguucgacagcacuc.............................................................................................................................................................................................................    3      1    JCK-3
......................uggaucuuguucgacagcacuA.............................................................................................................................................................................................................    61     1    JCK-2
......................uggaucuuguucAacagcacuc.............................................................................................................................................................................................................    1      1    JCK-1
......................uggCucuuguucgacagcacuc.............................................................................................................................................................................................................    1      1    LCK-3
......................uggaucuuguuNgacagcacuc.............................................................................................................................................................................................................    29     1    JCK-1
......................uggaucuCguucgacagcacuc.............................................................................................................................................................................................................    1      1    LT-1
......................uggaucuuguucgacagcacuA.............................................................................................................................................................................................................    67     1    JCK-3
......................uAgaucuuguucgacagcacuc.............................................................................................................................................................................................................    1      1    LT-3
```

```
                      Mature                                                                                        Star
uccaucuuguucguuuacccucuggaucuuguucgacagcacucuccuauauuuugccauuuaucuuuaaaugcgaaaucuuuaaaugcaaaauggcgacgaugauaggagugcugucgacaugauccagaaggugcagucgaauaagauacagagggaccacccacaguaccuacucucaacucucaucgcucgcaaucuuauucuucuccucacuuauuauuuuucagaucauuuucuuuuaaucuau
......................uggaucuuguucAacagcacuc..............................................................................................................................................................................................................   1      1   LT-1
......................uggaucuuguucgCcagcacuc..............................................................................................................................................................................................................   29     1   JCK-2
......................uggaucuugCucgacagcacuc..............................................................................................................................................................................................................   5      1   JCK-2
......................uggaCcuuguucgacagcacuc..............................................................................................................................................................................................................   1      1   LT-1
......................uggaucuuguucgacagcacuU..............................................................................................................................................................................................................   120    1   JCK-2
......................uUgaucuuguucgacagcacuc..............................................................................................................................................................................................................   1      1   LCK-3
......................uUgaucuuguucgacagcacuc..............................................................................................................................................................................................................   1      1   JCK-2
......................uggaucuuguucgacagcacuU..............................................................................................................................................................................................................   35     1   LT-3
......................uggaucuuguucgacGgcacuc..............................................................................................................................................................................................................   1      1   LT-1
......................uggaucuuguucgacaAcacuc..............................................................................................................................................................................................................   1      1   JCK-1
......................uggaucuuguucgCcagcacuc..............................................................................................................................................................................................................   2      1   LT-1
......................uggaucuuguucgacagcacuc..............................................................................................................................................................................................................   1033   0   LT-2
......................uggaucuuguucAacagcacuc..............................................................................................................................................................................................................   1      1   JCK-2
......................uUgaucuuguucgacagcacuc..............................................................................................................................................................................................................   1      1   JCK-1
......................uggaucuuguucgacagcCcuc..............................................................................................................................................................................................................   1      1   JCK-3
......................uggaucuuguucgacagcCcuc..............................................................................................................................................................................................................   1      1   LT-3
......................uggaucuuguucgaUagcacuc..............................................................................................................................................................................................................   42     1   JCK-2
......................uggaucuuguucgacagcacCc..............................................................................................................................................................................................................   2      1   JCK-2
......................uggCucuuguucgacagcacuc..............................................................................................................................................................................................................   2      1   JT-1
......................uUgaucuuguucgacagcacuc..............................................................................................................................................................................................................   1      1   LT-1
......................uAgaucuuguucgacagcacuc..............................................................................................................................................................................................................   2      1   LT-1
......................uggauUuuguucgacagcacuc..............................................................................................................................................................................................................   4      1   JCK-2
......................uggaucuugCucgacagcacuc..............................................................................................................................................................................................................   4      1   JCK-3
......................uggaucuuguucgUcagcacuc..............................................................................................................................................................................................................   3      1   JCK-2
......................uAgaucuuguucgacagcacuc..............................................................................................................................................................................................................   3      1   JCK-3
......................uggaucuuUuucgacagcacuc..............................................................................................................................................................................................................   3      1   JCK-2
......................uggaucuuguucgacagcacuU..............................................................................................................................................................................................................   27     1   LT-1
......................Cggaucuuguucgacagcacuc..............................................................................................................................................................................................................   2      1   JCK-1
......................uggaucuuguucgacagcCcuc..............................................................................................................................................................................................................   1      1   LT-2
......................uggGucuuguucgacagcacuc..............................................................................................................................................................................................................   2      1   JCK-2
......................uggaucuuguucgacaCcacuc..............................................................................................................................................................................................................   1      1   LT-1
......................uggaucuuguucgacagcacuU..............................................................................................................................................................................................................   63     1   JCK-1
......................uggaucuuguucgacaAcacuc..............................................................................................................................................................................................................   1      1   LT-3
......................uggaucuuguucgacagcacuc..............................................................................................................................................................................................................   69     0   JT-3
......................uggaucuuguucgacagcaUuc..............................................................................................................................................................................................................   10     1   JCK-3
......................uggaucuuguucgCcagcacuc..............................................................................................................................................................................................................   11     1   JCK-3
......................uggaucuuguucUacagcacuc..............................................................................................................................................................................................................   2      1   JCK-3
......................uggaucuuUuucgacagcacuc..............................................................................................................................................................................................................   3      1   JCK-3
......................uNgaucuuguucgacagcacuc..............................................................................................................................................................................................................   9      1   JCK-3
......................ugAaucuuguucgacagcacuc..............................................................................................................................................................................................................   2      1   JCK-1
......................uggaucuuguucgCcagcacuc..............................................................................................................................................................................................................   1      1   LT-2
......................ugAaucuuguucgacagcacuc..............................................................................................................................................................................................................   4      1   JCK-2
......................uggaucuuAuucgacagcacuc..............................................................................................................................................................................................................   1      1   JCK-3
......................Cggaucuuguucgacagcacuc..............................................................................................................................................................................................................   1      1   LT-2
......................uggaucuuguucgacagGacuc..............................................................................................................................................................................................................   2      1   JCK-2
......................Cggaucuuguucgacagcacuc..............................................................................................................................................................................................................   3      1   JCK-3
......................ugAaucuuguucgacagcacuc..............................................................................................................................................................................................................   5      1   LT-3
......................uAgaucuuguucgacagcacuc..............................................................................................................................................................................................................   1      1   LCK-3
......................uggauUuuguucgacagcacuc..............................................................................................................................................................................................................   3      1   JCK-1
......................uggaucuuguucgacCgcacuc..............................................................................................................................................................................................................   5      1   JCK-3
......................uggaucuuguucgacGgcacuc..............................................................................................................................................................................................................   2      1   LT-2
......................uggaucuuguucgaUagcacuc..............................................................................................................................................................................................................   1      1   LT-3
......................uggaucuuguucgacaUcacuc..............................................................................................................................................................................................................   4      1   JCK-2
......................uggaucuuguucgacagcacuU..............................................................................................................................................................................................................   25     1   LT-2
......................uggaucuuguucgacCgcacuc..............................................................................................................................................................................................................   3      1   JCK-1
......................uggCucuuguucgacagcacuc..............................................................................................................................................................................................................   19     1   JCK-1
......................uggaucuuguucgacagcacuN..............................................................................................................................................................................................................   4      1   JCK-1
......................uggaucuuguucgacagcacuNu.............................................................................................................................................................................................................   2      1   JCK-1
......................uggaucuuguucgacagcacucu.............................................................................................................................................................................................................   1104   0   JCK-3
......................uggCucuuguucgacagcacucu.............................................................................................................................................................................................................   1      1   JT-1
......................Cggaucuuguucgacagcacucu.............................................................................................................................................................................................................   1      1   JCK-1
......................uNgaucuuguucgacagcacucu.............................................................................................................................................................................................................   1      1   JCK-2
......................uggaucuuguucgacagcacuAu.............................................................................................................................................................................................................   2      1   JCK-3
......................uggaucuuguucgacagcacucA.............................................................................................................................................................................................................   18     1   LCK-3
......................uggaucuuguucgacagcacucu.............................................................................................................................................................................................................   15     0   LCK-3
......................uggaucuuguucgacagcaUucu.............................................................................................................................................................................................................   1      1   JCK-1
......................uggaucuuguucgacagcacCcu.............................................................................................................................................................................................................   1      1   JCK-2
......................uggaucuuguucgacagcacucu.............................................................................................................................................................................................................   4      0   JT-1
......................uggaucuuguucgCcagcacucu.............................................................................................................................................................................................................   1      1   JCK-1
......................uggaucuuCuucgacagcacucu.............................................................................................................................................................................................................   1      1   JCK-3
```

```
                      Mature                                                                            Star
uccaucuuguucguuuacccucuggaucuuguucgacagcacucuccuauauuuugccauuuaucuuuaaaugcgaaaucuuuaaaugcaaaauggcgacgaugauaggagugcugucgacaugauccagaaggugcagucgaauaagauacagagggaccacccacaguaccuacucucaacucucaucgcucgcaaucuuauucuucuccucacuuauuauuuuucagaucauuuucuuuuaaucuau
......................uggaucuuguucgacagcacucG.............................................................................................................................................................................................................	5	1	LT-2
......................uggaucuuguucgacagcacuAu.............................................................................................................................................................................................................	2	1	JCK-2
......................uggaucuuguucgaAagcacucu.............................................................................................................................................................................................................	1	1	LT-1
......................uNgaucuuguucgacagcacucu.............................................................................................................................................................................................................	2	1	JCK-1
......................uggaucuuguucgacagcacuUu.............................................................................................................................................................................................................	5	1	JT-3
......................uggaucuuguucgacagcacucA.............................................................................................................................................................................................................	64	1	JCK-2
......................uggaucuuguucgacagcacucA.............................................................................................................................................................................................................	8	1	LT-2
......................uggaucuuguucgacagcacucu.............................................................................................................................................................................................................	15	0	JT-2
......................uggaucuuguucgacagcacucu.............................................................................................................................................................................................................	14	0	LT-1
......................uggaucuuguucgCcagcacucu.............................................................................................................................................................................................................	2	1	LT-3
......................uggaucuuguucgacagcacuUu.............................................................................................................................................................................................................	50	1	LCK-3
......................uggaCcuuguucgacagcacucu.............................................................................................................................................................................................................	1	1	JCK-2
......................uggaucuuguucgacagcacucu.............................................................................................................................................................................................................	15	0	LT-2
......................uggCucuuguucgacagcacucu.............................................................................................................................................................................................................	2	1	JCK-1
......................uggaucuuguuNgacagcacucu.............................................................................................................................................................................................................	3	1	JCK-1
......................uggaucuuguucgacagcacucA.............................................................................................................................................................................................................	14	1	LT-1
......................uggaucuuguucgacagcacuAu.............................................................................................................................................................................................................	1	1	JCK-1
......................uggaucuuguucgacagcacucN.............................................................................................................................................................................................................	1	1	JCK-3
......................uggCucuuguucgacagcacucu.............................................................................................................................................................................................................	2	1	LT-2
......................uggaucuuguucgacagcacuUu.............................................................................................................................................................................................................	17	1	LCK-2
......................uggaucuuguucgaUagcacucu.............................................................................................................................................................................................................	5	1	JCK-1
......................uggaucuuguucgacagcacuUu.............................................................................................................................................................................................................	52	1	JCK-1
......................uggaucuuguucgacagcacucG.............................................................................................................................................................................................................	4	1	JCK-2
......................uggaucuuguucgacagcacuUu.............................................................................................................................................................................................................	32	1	LCK-1
......................uggaucuuguucgacagcacucG.............................................................................................................................................................................................................	1	1	JCK-3
......................uggaucuuguucgacagcacucu.............................................................................................................................................................................................................	19	0	JT-3
......................uggaucuuguucgacagcacuAu.............................................................................................................................................................................................................	1	1	LT-3
......................uggaucuuguucgacagcacucu.............................................................................................................................................................................................................	1493	0	JCK-2
......................uAgaucuuguucgacagcacucu.............................................................................................................................................................................................................	1	1	JCK-3
......................uggaucuuguucgacagcacucG.............................................................................................................................................................................................................	1	1	LT-3
......................uggaucuuguucgacagcacuUu.............................................................................................................................................................................................................	52	1	JCK-3
......................uggaucuuguucgacCgcacucu.............................................................................................................................................................................................................	1	1	JCK-3
......................ugAaucuuguucgacagcacucu.............................................................................................................................................................................................................	1	1	JCK-1
......................uggaucuuguucgacagcacuGu.............................................................................................................................................................................................................	1	1	LT-3
......................uggNucuuguucgacagcacucu.............................................................................................................................................................................................................	2	1	JCK-1
......................uggaucuuguucgacagcacuUu.............................................................................................................................................................................................................	20	1	LT-3
......................uggCucuuguucgacagcacucu.............................................................................................................................................................................................................	3	1	JCK-3
......................uggaucuuguucgacagcacuUu.............................................................................................................................................................................................................	22	1	LT-2
......................uggaucuuguucAacagcacucu.............................................................................................................................................................................................................	1	1	JCK-3
......................uggaucuuguucgCcagcacucu.............................................................................................................................................................................................................	2	1	JCK-2
......................uggaucuuguucgCcagcacucu.............................................................................................................................................................................................................	3	1	JCK-3
......................uggaucuuguucgacagUacucu.............................................................................................................................................................................................................	1	1	JCK-2
......................uggaucuuguucgacagcacucu.............................................................................................................................................................................................................	27	0	LT-3
......................uggaucuuguucgacagcacucA.............................................................................................................................................................................................................	52	1	JCK-3
......................uNgaucuuguucgacagcacucu.............................................................................................................................................................................................................	1	1	JCK-3
......................uggaucuuguucgacagcaNucu.............................................................................................................................................................................................................	1	1	JCK-1
......................uggaucuuguucgacagcacuUu.............................................................................................................................................................................................................	8	1	JT-2
......................ugAaucuuguucgacagcacucu.............................................................................................................................................................................................................	1	1	JCK-3
......................uggaucuuguucgaUagcacucu.............................................................................................................................................................................................................	2	1	JCK-3
......................uggaucuuguucgacagcacucA.............................................................................................................................................................................................................	59	1	JCK-1
......................uggaucCuguucgacagcacucu.............................................................................................................................................................................................................	1	1	JCK-2
......................uggaucuuguucgacagcacuGu.............................................................................................................................................................................................................	2	1	JCK-1
......................uggaucuuguucgacagcacucA.............................................................................................................................................................................................................	11	1	LT-3
......................uggaucuuNuucgacagcacucu.............................................................................................................................................................................................................	1	1	JCK-3
......................uggaucuuguucgacagcacuUu.............................................................................................................................................................................................................	21	1	LT-1
......................uggaucuuguucgaUagcacucu.............................................................................................................................................................................................................	2	1	JCK-2
......................ugUaucuuguucgacagcacucu.............................................................................................................................................................................................................	1	1	JCK-2
......................uggCucuuguucgacagcacucu.............................................................................................................................................................................................................	3	1	JCK-2
......................uggaucuuguucgacagcacuUu.............................................................................................................................................................................................................	70	1	JCK-2
......................uggaucuuguucgacagcacucu.............................................................................................................................................................................................................	860	0	JCK-1
......................uggaucuuguucgacagcacuAu.............................................................................................................................................................................................................	3	1	LT-1
......................uggaucuuguucgacagcacucuc............................................................................................................................................................................................................	11	0	JCK-1
......................uggaucuuguucgacagcacucuA............................................................................................................................................................................................................	31	1	JCK-2
......................uggaucuuguucgacagcacucuG............................................................................................................................................................................................................	1	1	JCK-1
......................uggaucuuguucgacagcacucuc............................................................................................................................................................................................................	10	0	JCK-3
......................uggaucuuguucgacagcacucuA............................................................................................................................................................................................................	22	1	JCK-3
......................uggaucuuguucgacagcacuUuc............................................................................................................................................................................................................	2	1	JCK-3
......................uggaucuuguucgacagcacuGuc............................................................................................................................................................................................................	3	1	JCK-2
......................uggaucuuguucgacagcacucuc............................................................................................................................................................................................................	5	0	LT-3
......................uggaucuuguucgacagcacucuU............................................................................................................................................................................................................	10	1	JCK-3
```

Mature (uggaucuuguucgacagcacu) — **Star** (gugcugucgacaugauccaga)

uccaucuuguucguuuacccucuggaucuuguucgacagcacucuccuauauuuugccauuuaucuuuaaaugcgaaaucuuuaaaugcaaaauggcgacgaugauaggagugcugucgacaugauccagaaggugcagucgaauaagauacagagggaccacccacaguaccuacucucaacucucaucgcucgcaaucuuauucuucuccucacuuauuauuuuucagaucauuuucuuuuaaucuau

uggaucuuguucgacagcacucuA	17	1	JCK-1
uggaucuuguucgacagcacucuA	3	1	LT-3
uggaucuuguucgacagcacucuc	10	0	JCK-2
uggaucuuguucgacagcacucuU	3	1	LT-2
uggaucuuguucgacagcacuAuc	1	1	JCK-2
uggaucuuguucgacagcacucuA	1	1	LT-2
uggaucuuguucgacagcacucuU	12	1	JCK-1
uggaucuuguucgacagcacucuc	6	0	LT-2
uggaucuuguucgacagcacuUuc	5	1	LCK-3
uggaucuuguucgacagcacucuU	1	1	LT-1
uggaucuuguuNgacagcacucuc	1	1	JCK-1
uggaucuuguucgacagcacucuc	7	0	LT-1
uggaucuuguucgacagcacucuU	13	1	JCK-2
uggaucuuguucgacagcacucuA	1	1	LCK-3
uggaucuuguucgacagcacuUuc	6	1	JCK-2
uggaucuuguucgacagcacuUuc	2	1	JCK-1
uggaucuuguucgacagcacucucc	8	0	LT-3
uggaucuuguucgacagcacucucc	6	0	JCK-3
uggaucuuguucgacagcacucuNc	1	1	LT-2
uggaucuuguucgacagcacucucc	17	0	LCK-1
uggaucuuguucgacagcacucucc	28	0	LCK-3
uggaucuuguucgacagcacucucc	18	0	LT-2
uggaucuuguucgacagcacucucc	5	0	JCK-2
uggaucuuguucgacagcacucucc	17	0	LT-1
uggaucuuguucgacagcaNucucc	1	1	JCK-1
uggaucuuguucgacagcacucucA	1	1	LT-3
uggaucuuguucgacagcacucucc	9	0	JCK-1
uggaucuuguucgacagcacucucc	15	0	LCK-2
uggaucuuguucgacagcacucuccu	1	0	JCK-1
uggaucuuguucgacagcacucuccC	1	1	LT-3
uggaucuuguucgacagcacucuccu	3	0	LT-1
uggaucuuguucgacagcacucucAu	1	1	LCK-3
uggaucuuguucgacagcacucucGu	1	1	JCK-2
uggaucuuguucgacagcacucuccA	1	1	LT-3
uggaucuuguucgacagcacucuccu	8	0	LT-3
uggaucuuguucgacagcacucuccu	24	0	LCK-3
uggaucuuguucgacagcacucucGu	1	1	JCK-3
uggaucuuguucgacagcacucuccu	9	0	LT-2
uggaucuuguucgacagcacucuccuU	1	1	LT-2
uggaucuuguucgacagcacucuccua	2	0	LT-2
ggaucuuguucgacagca	5	0	LT-2
ggaucuuguucgacagca	2	0	JCK-1
ggaucuuguucgacagca	10	0	JT-1
ggaucuuguucgacagcacu	1	0	LT-1
Ugaucuuguucgacagcacu	1	1	LT-1
ggaucuuguucgacagcacu	1	0	LT-2
ggaucuuguucgacagcacu	5	0	JCK-3
ggaucuuguucgacagcacu	2	0	JCK-1
ggaucuuguucgacagcacu	1	0	JCK-2
ggaucuuguucgacagcacuc	3	0	JCK-2
ggaucuuguucgacagcacuc	1	0	LT-1
ggaucuuguucgacagcacuc	2	0	JCK-3
ggaucuuguucgacagcacucu	2	0	JCK-3
ggaucuuguucgacagcacucu	1	0	JCK-2
ggaucuuguucgacagcacucuccu	2	0	LCK-3
ggaucuuguucgacagcacucuccuU	1	1	LT-1
gaucuuguucgacagcacu	2	0	JCK-1
gaucuuguucgacagcacu	2	0	JCK-3
gaucuuguucgacagcacucu	1	0	JCK-3
gaucuuguucgacagcacucuc	3	0	JCK-3
gaucuuguucgacagcacucuc	2	0	JCK-2
aucuuguucgacagcacu	1	0	LT-3
aucuuguucgacagcacuc	3	0	JCK-3
aucuuguucgacagcacuc	2	0	JCK-2
aucuuguucgacagcacuc	2	0	JCK-1
aucuuguucgacagcacuc	1	0	LT-3
aucuuguucgacagcacucu	1	0	LT-3
aucuuguucgacagcacuUu	2	1	JCK-3
aucuuguucgacagcacucu	1	0	LT-1
aucuuguucgacagcacucu	2	0	JCK-2

```
                      Mature                                                                                  Star
uccaucuuguucguuuacccucuggaucuuguucgacagcacucuccuauauuuugccauuuaucuuuaaaugcgaaaucuuuaaaugcaaaauggcgacgaugauaggagugcugucgacaugauccagaaggugcagucgaauaagauacagagggaccacccacaguaccuacucucaacucucaucgcucgcaaucuuauucuucuccucacuuauuauuuucagaucauuuucuuuuaaucuau
.........................aucuuguucgacagcacucuc    2   0   LT-3
.........................aucuuguucgacagcacucuc    2   0   JCK-1
.........................aucuuguucgacagcacucuc    2   0   LT-1
.........................aucuuguucgaNagcacucuc    1   1   JCK-1
.........................aucuuguucgacagcacucuc    1   0   JCK-2
.........................aucuuguucgacagcacucuc    1   0   JCK-3
.........................aucuuguucgacagcacucucc   3   0   LT-2
.........................aucuuguucgacagcacucucc   1   0   JCK-2
.........................aucuuguucgacagcacucucc   1   0   JCK-1
.........................aucuuguucgacagcacucucc   1   0   JCK-3
.........................aucuuguucgacagcacucucUu  1   1   JCK-1
..........................ucuuguucgacagcacuc      2   0   LT-3
..........................ucuuguucgacagcacuc      1   0   LT-1
..........................ucuuguucgacagcacucu     1   0   LT-3
..........................ucuuguucgacagcacucuc    1   0   JCK-3
..........................ucuuguucgacagcacucuccu  1   0   JCK-2
..........................ucuuguucgacagcacucuccu  1   0   LT-3
..........................ucuuguucgacagcacucuccu  1   0   LT-1
.............................................................................................uggcgacgaugauaggagugcug          9   0   LCK-3
........................................................................................................auaggagugcugucgacaugaucca      2   0   LT-2
........................................................................................................auaggagugcugucgacaugauccagaa   1   0   LT-1
.........................................................................................................uaggagugcugucgacaugauccaga     6   0   LCK-3
.........................................................................................................uaggagugcugucgacaugauccaga     1   0   LT-1
..........................................................................................................aggagugcugucgacaug            2   0   LT-2
..........................................................................................................aggagugcugucgacaug            4   0   LCK-3
..........................................................................................................aggagugcugucgacauga           1   0   LT-1
..........................................................................................................aggagugcugucgacaugau          8   0   LT-2
..........................................................................................................aggagugcugucgacaugau          6   0   LT-3
..........................................................................................................aggagugcugucgacaugauU         1   1   LT-1
..........................................................................................................aggagugcugucgacaugauU         2   1   LT-2
..........................................................................................................aggagugcugucgacaugauc         77  0   LT-2
..........................................................................................................aggagugcugucgacaugCuc         1   1   LT-2
..........................................................................................................aggagugcugucgacaugauc         19  0   LT-1
..........................................................................................................aggagugcugucgCcaugauc         1   1   LT-1
..........................................................................................................aggagugcugucgacaugauc         33  0   LT-3
..........................................................................................................aggagugcugucgacaugauU         1   1   LT-3
..........................................................................................................aggagugcugucgCcaugauc         1   1   LT-2
..........................................................................................................Cggagugcugucgacaugauc         1   1   LT-3
..........................................................................................................aggagugcugucgacaugauc         49  0   LCK-3
..........................................................................................................aggagugcugucgacaugaucc        1   0   LT-1
..........................................................................................................aggagugcugucgacaugauccag      3   0   LCK-3
..........................................................................................................Gggagugcugucgacaugauccagaaggug  1   1   JCK-3
...........................................................................................................ggagugcugucgacaugauc         2   0   LT-3
...........................................................................................................ggagugcugucgacaugaucc        1   0   LT-3
...........................................................................................................ggagugcugucgacaugaucc        2   0   LT-2
...........................................................................................................ggagugcugucgacaugaucc        9   0   LCK-3
...........................................................................................................ggagugcugucgacaugaucca       1   0   LT-3
...........................................................................................................ggGgugcugucgacaugauccag      2   1   JCK-1
...........................................................................................................ggGgugcugucgacaugauccag      1   1   JCK-3
...........................................................................................................ggGgugcugucgacaugauccag      3   1   JCK-2
...........................................................................................................ggagugcugucgacaugauccaga     8   0   LT-3
...........................................................................................................ggGgugcugucgacaugauccaga     15  1   JCK-2
...........................................................................................................ggGgugcugucgacaugauccaga     10  1   JCK-1
...........................................................................................................ggGgugcugucgacaugauccaga     20  1   JCK-3
...........................................................................................................ggagugcugucgacaugauccaga     4   0   LT-1
...........................................................................................................ggagugcugucgacaugauccaga     1   0   JCK-3
...........................................................................................................ggagugcugucgacaugauccaga     2   0   LT-2
...........................................................................................................ggagugcugucgacaugauccaga     2   0   JCK-2
...........................................................................................................ggagugcugucgacaugauccagaa    2   0   LT-2
...........................................................................................................ggagugcugucgacaugauccagaC    1   1   LT-1
............................................................................................................gagugcugucgacaugau          1   0   LT-3
............................................................................................................gagugcugucgacaugauc         3   0   LT-1
............................................................................................................gagugcugucgacaugauc         5   0   LT-3
............................................................................................................gagugcugucgacaugauc         1   0   LCK-3
............................................................................................................gagugcugucgacaugauc         1   0   LT-2
............................................................................................................gagugcugucgacaAgaucc        5   1   JT-2
............................................................................................................gagugcugucgacaAgaucc        10  1   JCK-3
............................................................................................................Aagugcugucgacaugaucc        1   1   LCK-3
............................................................................................................gagugcugucgacaugaucc        2   0   JCK-3
............................................................................................................gagugcugucgacaAgaucc        2   1   JCK-1
```

	Mature / Star		
	uccaucuuguucguuuacccucuggaucuuguucgacagcacucuccuauauuuugccauuuaucuuuaaaugcgaaaucuuuaaaugcaaaauggcgacgaugauaggagugcugucgacaugauccagaaggugcagucgaauaagauacagagggaccacccacaguaccuacucucaacucucaucgcucgcaaucuuauucuucuccucacuuauuauuuuucagaucauuuucuuuuaaucuau		
...gagugcugucgacaugaucc...	8	0	LCK-3
...gagugcugucgacaugaucc...	3	0	LT-2
...gagugcugucgacaugaucc...	7	0	LT-1
...gagugcugucgacaAgaucc...	8	1	JT-3
...gagugcugucgacaugaucc...	12	0	LT-3
...gagugcugucgacaAgaucc...	9	1	JCK-2
...gagugcugucgacaugaucU...	1	1	LT-1
...gagugcugucgacaugaucA...	1	1	JCK-1
...gagugcugucgacaAgaucca...	8	1	JCK-1
...gagugcugucgacaAgaucca...	15	1	JCK-2
...gagugcugucgacaugaucca...	22	0	LCK-2
...gagugcugucgacaugaucca...	4	0	LT-3
...gagugcugucgacaugauccC...	2	1	JCK-2
...gagugcugucgacaAgaucca...	8	1	JCK-3
...gCgugcugucgacaugaucca...	1	1	LCK-1
...gagugcugucgacaugaucca...	27	0	LCK-1
...gagugcugucgacaugaucca...	14	0	LT-2
...gagugcugucgacaugaucca...	3	0	LT-1
...gagugcugucgacaugauccag...	2	0	LCK-3
...gagugcugucgacaugauccag...	11	0	LT-1
...gagugcugucgacaugauccag...	14	0	LT-3
...gagugcugucgacaugauccag...	3	0	LT-2
...gagugcugucgacaAgauccaga...	44	1	JCK-3
...gagugcugucgacaAgauccaga...	72	1	JCK-2
...gagugcugucgacaugauccaga...	5	0	LT-1
...gagugcugucgacaugauccaga...	2	0	LT-2
...gagugcugucgacaAgauccaga...	35	1	JCK-1
....agugcugucgacaugauc...	4	0	LT-3
....agugcugucgacaugauc...	5	0	LT-2
....Ggugcugucgacaugauc...	1	1	JCK-3
....agugcugucgacaugauc...	4	0	LT-1
....Ggugcugucgacaugauc...	2	1	JCK-2
....agugcugucgacaugauc...	1	0	JCK-3
....agugcugucgacaugauc...	3	0	JCK-1
....agugcugucgacaugaucc...	4	0	LCK-3
....agugcugucgacaAgaucc...	3	1	JCK-3
....agugcugucgacaugaucc...	3	0	LT-3
....agugcugucgacaugaucc...	1	0	JCK-3
....Ggugcugucgacaugaucc...	1	1	JCK-1
....Ggugcugucgacaugaucc...	2	1	JCK-2
....agugcugucgacaugaucc...	1	0	LT-1
....agugcugucgacaAgaucc...	7	1	JCK-2
....agugcugucgacaugaucca...	2	0	LT-2
....agugcugucgacaAgaucca...	2	1	JCK-1
....agugcugucgacaAgaucca...	4	1	JCK-3
....agugcugucgacaugaucca...	3	0	LT-3
....agugcugucgacaAgaucca...	9	1	JCK-2
....agugcugucgacaugaucca...	10	0	LCK-3
....agugcugucgacaugaucca...	2	0	LT-1
....agugcugucgacaAgauccag...	5	1	JCK-1
....agugcugucgacaAgauccag...	2	1	JCK-2
....agugcugucgacaugauccag...	6	0	LT-1
....agugcugucgacaugauccag...	1	0	JCK-1
....agugcugucgacaugauccag...	2	0	JCK-2
....agugcugucgacaugauccaC...	4	1	LCK-3
....agugcugucgacaugauccag...	8	0	LT-2
....agugcugucgacaugauccag...	3	0	JCK-3
....agugcugucgacaAgauccag...	4	1	JCK-3
....agugcugucgacaugauccag...	8	0	LT-3
....agugUugucgacaugauccaga...	1	1	LT-2
....agugcugucgacaugauccagU...	1	1	LT-3
....agugcugucgacaugauccaga...	34	0	LT-3
....agugcugucgacaugauccaga...	14	0	JCK-2
....agugcugucgacaugauccaga...	29	0	LT-2
....agugcugucgacaAgauccaga...	1	1	JCK-3
....agugcugucgacaugauccaga...	3	0	JCK-3
....Ggugcugucgacaugauccaga...	13	1	JCK-3
....agugcugucgacaAgauccaga...	5	1	JCK-1
....Ggugcugucgacaugauccaga...	14	1	JCK-1
....agugUugucgacaugauccaga...	1	1	LCK-3

```
                      Mature                                                                                        Star
uccaucuuguucguuuacccucuggaucuuguucgacagcacucuccuauauuuugccauuuaucuuuaaaugcgaaaucuuuaaaugcaaaauggcgacgaugauaggagugcugucgacaugauccagaaggugcagucgaauaagauacagagggaccacccacaguaccuacucucaacucucaucgcucgcaaucuuauucuucuccucacuuauuauuuuucagaucauuuucuuuuaaucuau
.............................................................................................................Cgugcugucgacaugauccaga.......................................................................................................................  1    1  JCK-3
.............................................................................................................agugcugucgacaugauccaga.......................................................................................................................  8    0  JCK-1
.............................................................................................................agugcugucgacaugauccaga.......................................................................................................................  35   0  LT-1
.............................................................................................................agNgcugucgacaugauccaga.......................................................................................................................  1    1  LT-2
.............................................................................................................Cgugcugucgacaugauccaga.......................................................................................................................  3    1  JCK-2
.............................................................................................................agugcugucgacaugauccaga.......................................................................................................................  16   0  LCK-3
.............................................................................................................agugcugucgacaAgauccaga.......................................................................................................................  8    1  JCK-2
.............................................................................................................Ggugcugucgacaugauccaga.......................................................................................................................  18   1  JCK-2
.............................................................................................................agugcugucgacaAgauccagaa......................................................................................................................  1    1  JCK-2
.............................................................................................................agugcugucgacaugauccagaag.....................................................................................................................  1    0  JCK-2
..............................................................................................................gugcugucgacaugaucc..........................................................................................................................  148  0  JCK-2
..............................................................................................................gugcugucgacaugaucc..........................................................................................................................  29   0  LT-1
..............................................................................................................gugcugucgacaugaucc..........................................................................................................................  24   0  LCK-3
..............................................................................................................gugcugucgacaugaucc..........................................................................................................................  44   0  LT-2
..............................................................................................................gugcugucgaAaugaucc..........................................................................................................................  1    1  JCK-2
..............................................................................................................gugcugucgacaugaucc..........................................................................................................................  100  0  JCK-1
..............................................................................................................gugcugucgacaugaucc..........................................................................................................................  17   0  LCK-1
..............................................................................................................gugcugucgacaugaucc..........................................................................................................................  63   0  LT-3
..............................................................................................................gugcugucgacaAgaucc..........................................................................................................................  42   1  JCK-2
..............................................................................................................gugcugucgacaAgaucc..........................................................................................................................  5    1  JT-3
..............................................................................................................gugcugucgaGaugaucc..........................................................................................................................  1    1  JCK-1
..............................................................................................................gugcugucgacaugaucc..........................................................................................................................  16   0  LCK-2
..............................................................................................................gugcugucgCcaugaucc..........................................................................................................................  1    1  JCK-2
..............................................................................................................gugcugucgacaAgaucc..........................................................................................................................  3    1  JT-2
..............................................................................................................Augcugucgacaugaucc..........................................................................................................................  1    1  JCK-1
..............................................................................................................gugcugucgacaAgaucc..........................................................................................................................  16   1  JCK-1
..............................................................................................................gugcugucgacaAgaucc..........................................................................................................................  18   1  JCK-3
..............................................................................................................gugcugucgacaugaucc..........................................................................................................................  54   0  JT-1
..............................................................................................................gugcugucgacaugaucc..........................................................................................................................  12   0  JT-3
..............................................................................................................gugcugucgacaugaucc..........................................................................................................................  14   0  JT-2
..............................................................................................................gugcugucgacaugaucc..........................................................................................................................  107  0  JCK-3
..............................................................................................................gNgcugucgacaugaucca.........................................................................................................................  1    1  LCK-1
..............................................................................................................gugcugucgacaugaucca.........................................................................................................................  29   0  JCK-1
..............................................................................................................gugcugucgacaugaucca.........................................................................................................................  29   0  LCK-3
..............................................................................................................gugcugucgacaugaucca.........................................................................................................................  21   0  LT-1
..............................................................................................................gugcugucgacaAgaucca.........................................................................................................................  11   1  JCK-1
..............................................................................................................gugcugucgacaugaucca.........................................................................................................................  19   0  LCK-1
..............................................................................................................gugcugucgacaugaucca.........................................................................................................................  50   0  JCK-2
..............................................................................................................gugcugucgacaugaucca.........................................................................................................................  25   0  JT-3
..............................................................................................................gugcugucgacaugaucca.........................................................................................................................  25   0  LCK-2
..............................................................................................................gugcugucgacaugCucca.........................................................................................................................  1    1  JCK-3
..............................................................................................................gugcugucgacaugaucca.........................................................................................................................  28   0  JCK-3
..............................................................................................................guNcugucgacaugaucca.........................................................................................................................  1    1  LT-2
..............................................................................................................gugcugucgacGugaucca.........................................................................................................................  1    1  JT-1
..............................................................................................................gugcugucgacaugGucca.........................................................................................................................  1    1  JT-1
..............................................................................................................gugcugucgacaugaucca.........................................................................................................................  32   0  LT-2
..............................................................................................................gugcugucgacaugaucca.........................................................................................................................  37   0  JT-1
..............................................................................................................gugcugucgacaAgaucca.........................................................................................................................  7    1  JT-1
..............................................................................................................gugcugucgacaAgaucca.........................................................................................................................  8    1  JT-3
..............................................................................................................gugcugucgacaugaucca.........................................................................................................................  31   0  JT-2
..............................................................................................................gugcugucgacaugNucca.........................................................................................................................  1    1  LCK-1
..............................................................................................................gugcugucgacaAgaucca.........................................................................................................................  27   1  JCK-2
..............................................................................................................gugcugucgacaAgaucca.........................................................................................................................  9    1  JCK-3
..............................................................................................................gugcugucgacaugaucca.........................................................................................................................  31   0  LT-3
..............................................................................................................gugcugucgacaAgaucca.........................................................................................................................  7    1  JT-2
..............................................................................................................gugcugucgacaugauccag........................................................................................................................  84   0  LCK-3
..............................................................................................................gugcugucgacaugauccaA........................................................................................................................  2    1  JCK-1
..............................................................................................................gugcugucgacaugauccag........................................................................................................................  84   0  JCK-3
..............................................................................................................gugcugucgacaugauccag........................................................................................................................  62   0  LT-3
..............................................................................................................gugcugucgacaugauccag........................................................................................................................  4    0  JT-1
..............................................................................................................gugcugucgacaAgauccag........................................................................................................................  32   1  JCK-2
..............................................................................................................gugUugucgacaugauccag........................................................................................................................  1    1  LCK-3
..............................................................................................................gugcugucgacaugauccaA........................................................................................................................  1    1  JCK-3
..............................................................................................................gugcugucgacaugauccag........................................................................................................................  10   0  JT-3
..............................................................................................................gugcugucgacaugauccag........................................................................................................................  39   0  LT-1
..............................................................................................................gugcugucgacaugauccag........................................................................................................................  4    0  JT-2
..............................................................................................................gugcugucgacaAgauccag........................................................................................................................  9    1  JT-3
..............................................................................................................gugcugucgacaAgauccag........................................................................................................................  9    1  JT-2
..............................................................................................................gugcugucgacaugauccCg........................................................................................................................  1    1  JCK-2
..............................................................................................................gugcugucgacaugauccag........................................................................................................................  27   0  LT-2
```

Mature / **Star**

uccaucuuguucguuuacccucuggaucuuguucgacagcacucuccuauauuuugccauuuaucuuuaaaugcgaaaucuuuaaaugcaaaauggcgacgaugauaggagugcugucgacaugauccagaaggugcagucgaauaagauacagagggaccacccacaguaccuacucucaacucucaucgcucgcaaucuuauucuucuccucacuuauuauuuuucagaucauuuucuuuuaaucuau

Augcugucgacaugauccag	1	1	JCK-2
gugcugucgacaugauccag	54	0	JCK-1
gugcugucgacaugauccaA	1	1	LT-2
gugcugucgacaAgauccag	7	1	JCK-1
gugcugucgacaAgauccag	11	1	JCK-3
gugcugucgacaugauccag	110	0	JCK-2
gugcugucgacaugauccaA	1	1	LT-3
gugUugucgacaugauccaga	2	1	LT-1
gugcugucgacaugauccaga	2107	0	JCK-1
gugcugucgacaAgauccaga	22	1	JT-3
gugcugucgacaugNuccaga	1	1	LCK-1
gugcugucgacCugauccaga	2	1	JCK-3
gugcugucgacaugauccaga	2674	0	JCK-3
gugUugucgacaugauccaga	1	1	LT-3
gCgcugucgacaugauccaga	1	1	JCK-2
gugcugucgacaugauccaga	48	0	LCK-2
gugcugucgacaugauccaNa	1	1	JCK-1
gugcugucgacaugauccaga	471	0	LT-2
gugcugucgacaugaGccaga	1	1	JCK-1
gugcugucgacaugCuccaga	2	1	JCK-2
gugcugucgacaugauccCga	1	1	LT-3
guUcugucgacaugauccaga	2	1	JCK-3
gugcugucgaGaugauccaga	1	1	JCK-1
gugcugucgacaAgauccaga	19	1	JT-1
gugcugucgacaugauccaAa	4	1	JCK-2
gugcugucgCcaugauccaga	6	1	JCK-1
gugcugucgacCugauccaga	1	1	JCK-2
gugcugucgacaugauccaga	23	0	JT-1
Augcugucgacaugauccaga	1	1	LT-3
Cugcugucgacaugauccaga	1	1	LT-3
gugcugucgaAaugauccaga	1	1	JCK-3
gugcugucgacaugauccagU	2	1	JCK-3
gugcugucgacaugauccagN	1	1	JCK-1
gugcugucgacaugauccGga	2	1	JCK-1
gugUugucgacaugauccaga	8	1	JCK-1
gugcugucgacCugauccaga	2	1	LT-2
gugcugucgacNugauccaga	5	1	JCK-1
gugcugucgacaAgauccaga	712	1	JCK-1
gugcugucNacaugauccaga	1	1	JCK-3
gugcugucgacauAauccaga	1	1	JCK-2
gugcugucgacaugCuccaga	2	1	LT-3
Cugcugucgacaugauccaga	1	1	LCK-3
gugcugucgCcaugauccaga	2	1	LT-2
gugcugucgacaugaucUaga	4	1	JCK-2
Augcugucgacaugauccaga	8	1	JCK-3
gugcugucgCcaugauccaga	2	1	JCK-3
gugcugucgacaugauccCga	3	1	JCK-1
Uugcugucgacaugauccaga	2	1	JCK-2
gugcugucgacaugauccaga	34	0	LCK-1
Cugcugucgacaugauccaga	1	1	JCK-1
gugcuguUgacaugauccaga	1	1	JCK-2
gugcugucgaAaugauccaga	1	1	LT-2
gugcugucgacaugauccaga	686	0	LT-3
gugcugucgacaAgauccaga	910	1	JCK-3
Augcugucgacaugauccaga	1	1	JCK-1
gugGugucgacaugauccaga	1	1	JCK-1
gugcugucgacaugauccaga	48	0	JT-2
Augcugucgacaugauccaga	1	1	LT-1
gugcugucgacaCgauccaga	1	1	JCK-2
gugcugucgacaugauccGga	1	1	LT-3
gugcugucgacaugauccaga	464	0	LT-1
gAgcugucgacaugauccaga	2	1	JCK-2
gugcugucgacaugauccCga	2	1	JCK-2
gugcugucgacaugauccaga	3681	0	JCK-2
guAcugucgacaugauccaga	1	1	JCK-3
gugcugucgacaugCuccaga	2	1	JCK-3
gNgcugucgacaugauccaga	1	1	JCK-2
gugcugucgacaugaGccaga	1	1	JCK-2
gugcugucgacaugaucUaga	1	1	JCK-3
gugcugucgacCugauccaga	4	1	JCK-1

Mature **Star**

```
uccaucuuguucguuuacccucuggaucuuguucgacagcacucuccuauauuuugccauuuaucuuuaaaugcgaaaucuuuaaaugcaaaauggcgacgaugauaggagugcugucgacaugauccagaaggugcagucgaauaagauacagagggaccacccacaguaccuacucucaacucucaucgcucgcaaucuuauucuucuccucacuuauuauuuuucagaucauuuucuuuuaaucuau
..............................................................................................................gugcugucgacaugauccaga.......................................................................................................................   74    0   JT-3
..............................................................................................................gugUugucgacaugauccaga.......................................................................................................................   9     1   JCK-3
..............................................................................................................Augcugucgacaugauccaga.......................................................................................................................   2     1   LCK-3
..............................................................................................................gugcugucgacaCgauccaga.......................................................................................................................   1     1   JCK-3
..............................................................................................................gugcugucgacCugauccaga.......................................................................................................................   1     1   LCK-2
..............................................................................................................gugcugucgacaAgauccaga.......................................................................................................................   18    1   JT-2
..............................................................................................................gugcugucgGcaugauccaga.......................................................................................................................   1     1   JCK-3
..............................................................................................................gugcugucgacaugauccagU.......................................................................................................................   1     1   JCK-2
..............................................................................................................gugcugucgacaugaucUaga.......................................................................................................................   2     1   JCK-1
..............................................................................................................gugcugucgacaugauccaUa.......................................................................................................................   2     1   JCK-3
..............................................................................................................gugcugucgacaugCuccaga.......................................................................................................................   2     1   JCK-1
..............................................................................................................gugcugucgCcaugauccaga.......................................................................................................................   1     1   LT-1
..............................................................................................................gNgcugucgacaugauccaga.......................................................................................................................   3     1   JCK-1
..............................................................................................................gugcugucgacaugauccaUa.......................................................................................................................   3     1   JCK-2
..............................................................................................................gNgcugucgacaugauccaga.......................................................................................................................   3     1   JCK-3
..............................................................................................................guAcugucgacaugauccaga.......................................................................................................................   2     1   LT-2
..............................................................................................................gugcugucgacaugauccaga.......................................................................................................................   315   0   LCK-3
..............................................................................................................gugcugucgacCugauccaga.......................................................................................................................   2     1   LT-1
..............................................................................................................Uugcugucgacaugauccaga.......................................................................................................................   1     1   LT-2
..............................................................................................................gugcugucgCcaugauccaga.......................................................................................................................   3     1   JCK-2
..............................................................................................................gugUugucgacaugauccaga.......................................................................................................................   9     1   JCK-2
..............................................................................................................gAgcugucgacaugauccaga.......................................................................................................................   1     1   JCK-1
..............................................................................................................gugcugucgacaugauccagG.......................................................................................................................   3     1   LCK-3
..............................................................................................................gugcugucgacaugauccagC.......................................................................................................................   1     1   LT-3
..............................................................................................................gugcugucgacaugaucGaga.......................................................................................................................   1     1   JCK-1
..............................................................................................................guNcugucgacaugauccaga.......................................................................................................................   2     1   LT-2
..............................................................................................................gAgcugucgacaugauccaga.......................................................................................................................   1     1   JCK-3
..............................................................................................................gugcugucgaAaugauccaga.......................................................................................................................   1     1   JCK-1
..............................................................................................................Augcugucgacaugauccaga.......................................................................................................................   5     1   JCK-2
..............................................................................................................gugcugucgacCugauccaga.......................................................................................................................   1     1   LCK-1
..............................................................................................................gugNugucgacaugauccaga.......................................................................................................................   3     1   JCK-1
..............................................................................................................gugcugucgacaAgauccaga.......................................................................................................................   1129  1   JCK-2
..............................................................................................................Augcugucgacaugauccaga.......................................................................................................................   1     1   LT-2
..............................................................................................................gugcugucAacaugauccagaa......................................................................................................................   5     1   JCK-2
..............................................................................................................gugcugucgacaAgauccagaa......................................................................................................................   7     1   JCK-2
..............................................................................................................gugcugucgacaugauccagaG......................................................................................................................   1     1   LT-1
..............................................................................................................gugcugucgacaugauccagaa......................................................................................................................   6     0   JCK-3
..............................................................................................................gugcugucgacaugauccagaa......................................................................................................................   4     0   JCK-1
..............................................................................................................gugcugucgacaugauccagaa......................................................................................................................   11    0   JCK-2
..............................................................................................................gugcugucgacaAgauccagaa......................................................................................................................   6     1   JCK-1
..............................................................................................................gugcugucgacaAgauccagaa......................................................................................................................   2     1   JCK-3
..............................................................................................................gugcugucgacaugauccagaa......................................................................................................................   3     0   LT-2
..............................................................................................................gugcugucgacaugauccagaaA.....................................................................................................................   1     1   JCK-1
..............................................................................................................gugcugucgacaugauccagaaU.....................................................................................................................   2     1   JCK-2
..............................................................................................................gugcugucgacaugauccagaaU.....................................................................................................................   1     1   JCK-1
..............................................................................................................gugcugucgacaugauccagaCg.....................................................................................................................   1     1   LT-1
..............................................................................................................gugcugucgacaugauccagaagg....................................................................................................................   2     0   LT-1
..............................................................................................................gugcugucgacaugauccagaaggu...................................................................................................................   2     0   JCK-2
...............................................................................................................ugcugucgacaugaucca.........................................................................................................................   2     0   LCK-3
...............................................................................................................ugcugucgacaugaucca.........................................................................................................................   6     0   LT-3
...............................................................................................................ugcugucgacaugaucca.........................................................................................................................   1     0   LT-1
...............................................................................................................ugcugucgacaugaucca.........................................................................................................................   5     0   LT-2
...............................................................................................................ugcugucgacaugaucca.........................................................................................................................   1     0   JCK-2
...............................................................................................................ugcugucgacaugauccaA........................................................................................................................   2     1   JCK-1
...............................................................................................................ugcugucgacaugauccaC........................................................................................................................   1     1   JCK-1
...............................................................................................................ugcugucgacaugauccag........................................................................................................................   1     0   JCK-3
...............................................................................................................ugcugucgacaugauccag........................................................................................................................   2     0   JCK-2
...............................................................................................................ugcugucgacaugauccaA........................................................................................................................   1     1   JCK-2
...............................................................................................................ugcugucgacaugauccag........................................................................................................................   2     0   LT-3
...............................................................................................................ugcugucgacaAgauccag........................................................................................................................   2     1   JCK-3
...............................................................................................................ugcugucgacaAgauccag........................................................................................................................   2     1   JCK-2
...............................................................................................................ugcugucgacaAgauccag........................................................................................................................   2     1   JCK-1
...............................................................................................................ugcugucgacaugauccaga.......................................................................................................................   10    0   JT-1
...............................................................................................................uNcugucgacaugauccaga.......................................................................................................................   1     1   JCK-2
...............................................................................................................ugcugucgacaAgauccaga.......................................................................................................................   20    1   JT-3
...............................................................................................................ugcugucgacaAgauccaga.......................................................................................................................   13    1   JT-2
...............................................................................................................ugcugucgacaugauccaga.......................................................................................................................   25    0   JCK-1
...............................................................................................................ugcugucgacaugauccaga.......................................................................................................................   1     0   JT-3
...............................................................................................................ugcugucgacaAgauccaga.......................................................................................................................   105   1   JCK-3
...............................................................................................................ugcugucgacaugauccaga.......................................................................................................................   51    0   JCK-2
```

Mature **Star**

uccaucuuguucguuuacccucuggaucuuguucgacagcacucuccuauauuuugccauuuaucuuuaaaugcgaaaucuuuaaaugcaaaauggcgacgaugauaggagugcugucgacaugauccagaaggugcagucgaauaagauacagagggaccacccacaguaccuacucucaacucucaucgcucgcaaucuuauucuucuccucacuuauuauuuucagaucauuuucuuuuaaaucuau			
ugcugucgacaugauccaga	17	0	LCK-2
ugcugucgacaAgauccaga	125	1	JCK-2
ugcugucgacaAgauccaga	22	1	JT-1
ugcugucgacaugauccaga	15	0	LT-1
ugcugucgacaugaucUaga	1	1	JCK-3
ugcugucgacaugauccaga	20	0	LT-3
ugcugucgacaugauccaga	24	0	LCK-1
ugUugucgacaugauccaga	1	1	JCK-2
ugcugucgacaugauccaga	29	0	JCK-3
ugcugucgacaugauccaga	29	0	LT-2
ugcugucgacaugauccaga	20	0	LCK-3
ugcugucgacaAgauccaga	79	1	JCK-1
ugcugucgacaugauccagaa	1	0	JCK-2
ugcugucgacaAgauccagaa	1	1	JCK-3
ugcugucgacaAgauccagaa	2	1	JCK-1
ugcugucgacaugauccagaC	2	1	LT-1
ugcugucgacaugauccagaC	1	1	JCK-2
ugcugucgacaugauccagaU	1	1	JCK-3
ugcugucgacaugauccagaag	1	0	LT-3
ugcugucgacaugauccagaag	1	0	LT-1
ugcugucgacaugauccagaaggu	1	0	JCK-3
ugcugucgacaAgauccagaaggu	1	1	JCK-2
gcugucgacaAgauccagaag	1	1	JCK-2
gcugucgacaAgauccagaag	3	1	JT-2
gcugucgacaAgauccagaag	3	1	JCK-3
gcugucgacaugauccagaag	2	0	JCK-2
gcugucgacaAgauccagaag	3	1	JCK-1
gcugucgacaugauccagaag	1	0	LT-1
gcugucgacaAgauccagaag	4	1	JT-3
cugucgacaAgauccaga	1	1	JCK-1
cugucgacaugauccaga	1	0	JCK-1
cugucgacaugauccaga	1	0	JCK-3
cugucgacaugauccaga	2	0	JT-3
cugucgacaugauccaga	1	0	JT-2
cugucgacaugauccaga	1	0	LT-1
cugucgacaugauccaga	2	0	LT-2
cugucgacaugauccaga	1	0	LT-3
cugucgacaAgauccaga	1	1	JCK-2
cugucgacaAgauccagaa	2	1	JCK-2
cugucgacaAgauccagaa	2	1	JCK-1
cugucgacaugauccagaa	1	0	JCK-2
cugucgacaugauccagaa	1	0	LT-1
cugucgacaugCuccagaa	1	1	JCK-2
cugucgacaAgauccagaa	1	1	JCK-3
cugucgacaugauccagaa	1	0	LT-2
cugucgacaugauccagaa	1	0	JCK-1
cugucgacaugauccagaa	6	0	LCK-3
cugucgacaugauccagaag	5	0	JCK-1
cugucgacaAgauccagaag	1	1	JCK-1
cugucgacaAgauccagaag	4	1	JCK-2
cugucgacaugauccagaag	1	0	LT-3
cugucgacaugauccagaag	6	0	JCK-2
cugucgacaugauccagaag	5	0	LT-1
cugucgacaugauccagaaU	1	1	LT-3
cugucgacaAgauccagaag	5	1	JCK-3
cugucgacaugauccagaag	2	0	JCK-3
cugucgacaugauccagaagg	1	0	JCK-2
cugucgacaugauccagaagg	1	0	JCK-3
cugucgacaugauccagaagU	3	1	JCK-1
cugucgacaAgauccagaagg	4	1	JCK-2
cugucgacaAgauccagaagg	5	1	JCK-1
cugucgacaugauccagaagU	1	1	LT-3
cugucgacaAgauccagaagg	3	1	JCK-3
ugucgacaAgauccagaagg	1	1	JCK-1
ugucgacaAgauccagaaggu	11	1	JCK-2
ugucgacaAgauccagaaggu	17	1	JCK-3
ugucgacaAgauccagaaggu	6	1	JCK-1
ugucgacaugauccagaaggu	1	0	LT-2
ucgacaAgauccagaaggugc	3	1	JCK-3
ucgacaAgauccagaaggugc	2	1	JCK-1

```
                      Mature                                                                                    Star
uccaucuuguucguuuacccucuggaucuuguucgacagcacucuccuauauuuugccauuuaucuuuaaaugcgaaaucuuuaaaugcaaaauggcgacgaugauaggagugcugucgacaugauccagaaggugcagucgaauaagauacagagggaccacccacaguaccuacucucaacucucaucgcucgcaaucuuauucuucuccucacuuauuauuuuucagaucauuuucuuuuaaucuau
....................................................................................................................ucgacaAgauccagaaggugc.................................................................................................................   1   1   JCK-2
```
